# PTER is a *N*-acetyltaurine hydrolase that regulates feeding and obesity

**DOI:** 10.1038/s41586-024-07801-6

**Published:** 2024-08-07

**Authors:** Wei Wei, Xuchao Lyu, Andrew L. Markhard, Sipei Fu, Rachel E. Mardjuki, Peter E. Cavanagh, Xianfeng Zeng, Jakub Rajniak, Nannan Lu, Shuke Xiao, Meng Zhao, Maria Dolores Moya-Garzon, Steven D. Truong, Jonathan Chiu‐Chun Chou, Lianna W. Wat, Saranya Chidambaranathan-Reghupaty, Laetitia Coassolo, Duo Xu, Fangfang Shen, Wentao Huang, Cuauhtemoc B. Ramirez, Cholsoon Jang, Lingyin Li, Katrin J. Svensson, Michael A. Fischbach, Jonathan Z. Long

**Affiliations:** 1 Department of Pathology, Stanford University School of Medicine, Stanford, CA USA; 2 Sarafan ChEM-H, Stanford University, Stanford, CA USA; 3 Wu Tsai Human Performance Alliance, Stanford University, Stanford, CA USA; 4 Department of Biology, Stanford University, Stanford, CA USA; 5 Department of Biochemistry, Stanford University, Stanford, CA USA; 6 Department of Chemistry, Stanford University, Stanford, CA USA; 7 Department of Bioengineering, Stanford University, Stanford, CA USA; 8 Department of Neurology and Neurological Sciences, Stanford University, Stanford, CA USA; 9 Wu Tsai Neurosciences Institute, Stanford University School of Medicine, Stanford, CA USA; 10 Stanford Diabetes Research Center, Stanford University School of Medicine, Stanford, CA USA; 11 Department of Biology, Massachusetts Institute of Technology, Cambridge, MA USA; 12 Cardiovascular Institute, Stanford University School of Medicine, Stanford, CA USA; 13 Department of Biological Chemistry, University of California Irvine, Irvine, CA USA; 14 Arc Institute, Palo Alto, CA USA; 15 The Phil and Penny Knight Initiative for Brain Resilience at the Wu Tsai Neurosciences Institute, Stanford University, Stanford, CA USA

**Keywords:** Hydrolases, Obesity

## Abstract

Taurine is a conditionally essential micronutrient and one of the most abundant amino acids in humans^1–3^. In endogenous taurine metabolism, dedicated enzymes are involved in the biosynthesis of taurine from cysteine and in the downstream metabolism of secondary taurine metabolites^4,5^. One taurine metabolite is *N*-acetyltaurine^6^. Levels of *N*-acetyltaurine are dynamically regulated by stimuli that alter taurine or acetate flux, including endurance exercise^7^, dietary taurine supplementation^8^ and alcohol consumption^6,9^. So far, the identities of the enzymes involved in *N*-acetyltaurine metabolism, and the potential functions of *N*-acetyltaurine itself, have remained unknown. Here we show that the body mass index associated orphan enzyme phosphotriesterase-related (PTER)^10^ is a physiological *N*-acetyltaurine hydrolase. In vitro, PTER catalyses the hydrolysis of *N*-acetyltaurine to taurine and acetate. In mice, PTER is expressed in the kidney, liver and brainstem. Genetic ablation of *Pter* in mice results in complete loss of tissue *N*-acetyltaurine hydrolysis activity and a systemic increase in *N*-acetyltaurine levels. After stimuli that increase taurine levels, *Pter* knockout mice exhibit reduced food intake, resistance to diet-induced obesity and improved glucose homeostasis. Administration of *N*-acetyltaurine to obese wild-type mice also reduces food intake and body weight in a GFRAL-dependent manner. These data place PTER into a central enzymatic node of secondary taurine metabolism and uncover a role for PTER and *N*-acetyltaurine in body weight control and energy balance.

## Abstract (web summary)

The orphan enzyme phosphotriesterase-related (PTER) is identified as a mammalian *N*-acetyltaurine hydrolase that has roles in regulating body weight and energy balance.

## Main

Taurine is a conditionally essential micronutrient and an abundant amino sulfonic acid that is found in mammalian tissues and many foods^2,11^. Levels of taurine are particularly high in excitable tissues such as the heart, eyes, brain and muscles^5^. Taurine has been described to have pleiotropic cellular and physiological functions, particularly in the context of metabolic homeostasis^11–13^. Genetic reduction of tissue taurine levels leads to muscle atrophy^14,15^, decreased exercise capacity^16^ and mitochondrial dysfunction in multiple tissues^14,17^. Conversely, taurine supplementation has been reported to reduce mitochondrial redox stress^11^, enhance exercise performance^18^ and suppress body weight^19^.

The biochemistry and enzymology of taurine metabolism has attracted considerable research interest. In the endogenous taurine biosynthesis pathway, cysteine is metabolized through cysteine dioxygenase (CDO) and cysteine sulfinic acid decarboxylase (CSAD) to generate hypotaurine^20,21^, which is subsequently oxidized by flavin-containing monooxygenase 1 (FMO1) to produce taurine^22^. In addition, cysteine can undergo an alternative pathway through cysteamine and cysteamine dioxygenase (ADO) to produce hypotaurine^23^. Downstream of taurine itself are several secondary taurine metabolites that include taurocholate, taurocyamine and *N*-acetyltaurine^4^. The only enzyme known to catalyse one of these downstream pathways is BAAT, which conjugates taurine to bile acyl-CoAs to produce taurocholate and other bile salts^24^. Beyond BAAT, the molecular identities of the additional enzymes that mediate secondary taurine metabolism have not yet been established.

*N*-acetyltaurine is a particularly interesting and poorly studied secondary taurine metabolite. Levels of *N*-acetyltaurine in biological fluids are dynamically regulated by diverse physiological perturbations that increase taurine and/or acetate flux, including endurance exercise^7,19^, alcohol consumption^6,9^ and nutritional taurine supplementation^19^. In addition, *N*-acetyltaurine exhibits chemical structural similarities with signalling molecules that include the neurotransmitter acetylcholine^25^ and the glucoregulatory long-chain *N*-fatty acyl taurines^26^, which suggests that it may also function as a signalling metabolite. However, the biosynthesis, degradation and potential functions of *N*-acetyltaurine remain unclear.

Using an activity-guided fractionation approach, here we identify PTER, an orphan enzyme of previously unknown function, as the principal mammalian *N*-acetyltaurine hydrolase. In vitro, recombinant PTER exhibits a narrow substrate range that is largely restricted to *N*-acetyltaurine. Genetic ablation of *Pter* in mice abolishes tissue *N*-acetyltaurine hydrolysis activities and results in concomitant increases in *N*-acetyltaurine across tissues. Last, using genetic clues linking the human *PTER* locus to body mass index (BMI), we provide functional evidence that genetic ablation of *Pter* in mice, or pharmacological administration of *N*-acetyltaurine, suppresses body weight and adiposity. The full anorexigenic and anti-obesity effects of *N*-acetyltaurine require functional GFRAL receptors. These data define a PTER-dependent pathway of taurine metabolism linked to energy balance.

## Purification of PTER from kidney

**Fig. 1:**
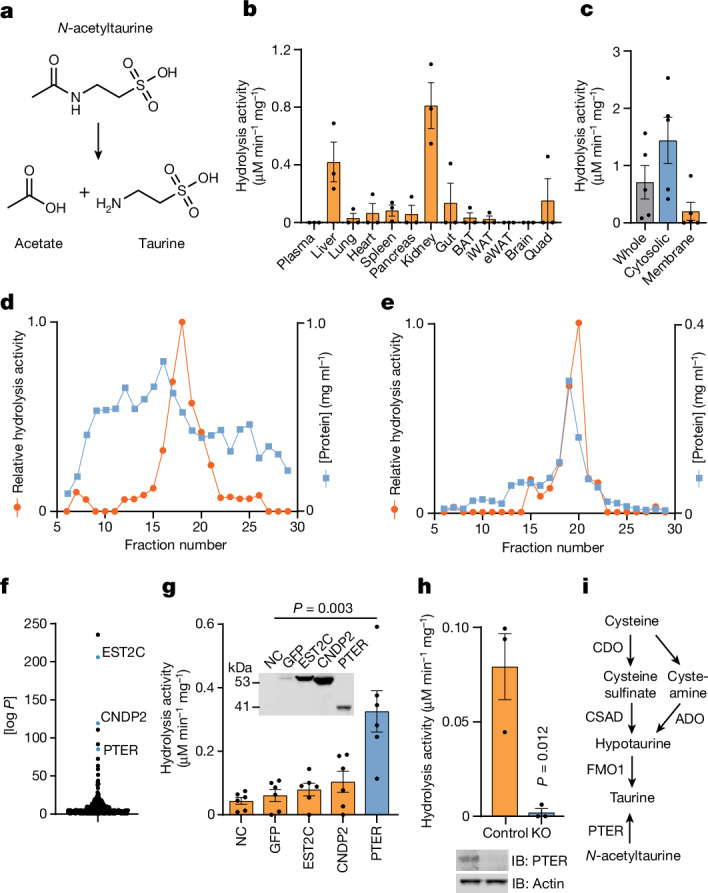
Activity-guided fractionation identifies PTER as a *N*-acetyltaurine hydrolase. **a**, Schematic showing the conversion of *N*-acetyltaurine to acetate and taurine. **b**, *N*-acetyltaurine hydrolysis activity of the indicated mouse whole-tissue homogenate. Tissue samples were collected from 10–14-week-old male C57BL/6J mice. Reactions were performed using 100 µg tissue homogenates at 37 °C for 1 h with 100 µM *N*-acetyltaurine. *N* = 3 per group. BAT, brown adipose tissue; eWAT, epididymal white adipose tissue; iWAT, inguinal white adipose tissue; Quad, quadriceps muscle. **c**, *N*-acetyltaurine hydrolysis activity in the indicated fraction of total kidney lysate. Reactions were performed as in **b**. *N* = 5 per group. **d**,**e**, Relative *N*-acetyltaurine hydrolysis activity and protein concentrations of the indicated fraction following anion-exchange chromatography (**d**) or size-exclusion chromatography (**e**). *N* = 1 per data point. **f**, Byonic *P* values of proteins identified in fraction 20 following size-exclusion chromatography. **g**,**h**, *N*-acetyltaurine hydrolysis activity from HEK293T cell lysates after transfection with the indicated plasmids (**g**, *N* = 6 per group) or from control or *PTER* KO cell lysates (**h**, *N* = 3 per group). Reactions were performed as in **b**. Western blots in **g** and **h** used an anti-Flag antibody of HEK293T cell lysates 2 days after the indicated transfection (**g**) or anti-PTER antibody in WT and *PTER* KO cells (**h**). IB, immunoblot. **i**, Schematic of revised taurine metabolic pathway showing the role of PTER as a *N*-acetyltaurine hydrolase. ADO, cysteamine deoxygenate; CDO, cysteine dioxygenase; CSAD, cysteine sulfinic acid decarboxylase; FMO1, flavin-containing monooxygenase 1. For **b**, **c**, **g** and **h**, data are shown as the mean ± s.e.m. For **h**, the loading control was performed on the same blot. In **g** and **h**, *P* values were calculated from two-tailed unpaired *t*-tests and not adjusted for multiple comparisons. All experiments were repeated twice and similar results were obtained. Source Data

To identify the enzyme (or enzymes) that regulates *N*-acetyltaurine, we used an in vitro enzyme activity-guided approach to detect and purify *N*-acetyltaurine hydrolysis activity products from mouse tissues (Fig. 1a). Total tissue homogenates were incubated with *N*-acetyltaurine (100 µM, 1 h, 37 °C) and the reaction was monitored by liquid chromatography–mass spectrometry (LC–MS). In kidneys, we observed strong tissue hydrolysis activity that resulted in the production of taurine and the depletion of *N*-acetyltaurine in the reaction (Fig. 1b). The liver also exhibited similar *N*-acetyltaurine hydrolysis activity, whereas little activity was detected in the other tissues tested (Fig. 1b). Centrifugation of kidney tissues into a cytosolic and a 100,000*g* membrane fraction revealed enrichment of this hydrolysis activity in the cytosol (Fig. 1c). Next, we fractionated total kidney cytosol fractions by anion-exchange chromatography, which revealed a single spike of enzyme activity that peaked in fractions 15–20 (Fig. 1d). The fractions with peak activity (17–19 inclusive) were pooled and then subjected to size-exclusion chromatography. Again, a single peak of activity was observed that centred around fraction 20 (Fig. 1e). The active fraction 20 was analysed by shotgun proteomics (Supplementary Table 1). Figure 1f shows the ranking of these proteins by Byonic *P* values, which measures the likelihood of protein identification by random chance. The highest-ranking enzymes within this list were the esterase EST2C (also known as CES2C; rank 2)^27^, the peptidase/synthase CNDP2 (rank 3)^28–30^ and PTER (rank 6), a putative metal-dependent hydrolase of unknown enzymatic activity or function.

We transfected cDNAs encoding each of these three enzymes into HEK293T cells. Lysates from cells transfected with PTER, but not EST2C or CNDP2, exhibited higher *N*-acetyltaurine hydrolysis activity compared with GFP-transfected control lysates (Fig. 1g). We also observed that GFP-transfected cells exhibited a basal *N*-acetyltaurine hydrolysis activity over background, which we speculated might be due to the endogenous human PTER. Indeed, cell lysates from HEK293T cells in which *PTER* was knocked out exhibited complete loss of this hydrolysis activity compared with control cells (Fig. 1h). We conclude that overexpression of PTER is sufficient to confer *N*-acetyltaurine hydrolysis activity to cell lysates. In addition, PTER is necessary for the endogenous *N*-acetyltaurine hydrolysis activity in HEK293T cells. Based on these data, Fig. 1i shows the new biochemical assignment of PTER as a *N*-acetyltaurine hydrolase within the context of the endogenous biochemical pathways of taurine metabolism.

## Enzymology and mutagenesis of PTER

**Fig. 2:**
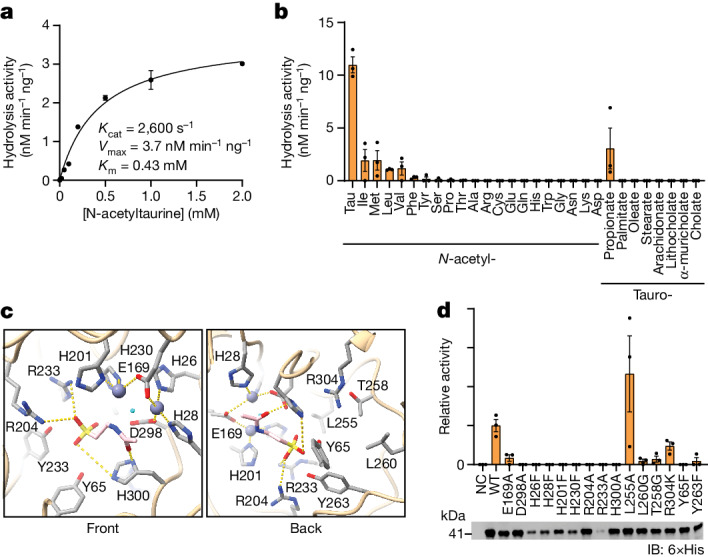
Enzymology and mutagenesis of recombinant mouse PTER in vitro. **a**,**b**, Hydrolysis rates following incubation of purified recombinant mouse PTER (100 ng) and the indicated concentration of *N*-acetyltaurine (**a**) or 100 µM of the indicated substrates (**b**). Reactions were performed for 1 h at 37 °C. *N* = 3 per group. **c**, Molecular docking of mouse PTER and *N*-acetyltaurine. Individual amino acid residues, two zinc ions (dark blue) and one water molecule (light blue) are highlighted. **d**, *N*-acetyltaurine hydrolysis activity of total bacterial lysates overexpressing the indicated mouse PTER mutant (top) and western blot using an anti-6×His antibody (bottom). Reactions were performed with 100 µM *N*-acetyltaurine for 1 h at 37 °C. *N* = 3 per group. For **a**, **b** and **d**, data are shown as the mean ± s.e.m. Data were fitted to Michaelis–Menten kinetics (solid line) using GraphPad Prism. All experiments were repeated twice and similar results were obtained. Source Data

To study the enzymology of PTER, we generated purified recombinant mouse PTER through heterologous expression in bacteria and determined its enzymatic activity using *N*-acetyltaurine as a substrate. Fitting enzyme activity data to Michaelis–Menton kinetics revealed that recombinant PTER exhibited the following values: *K*_cat_ = 2,600 s^–1^, *K*_m_ = 430 μM and *V*_max_ = 3.7 nM min^–1^ mg^–1^ (Fig. 2a). In a scan of potential substrates, PTER was most active when *N*-acetyltaurine was used (Fig. 2b). PTER also catalysed the hydrolysis of several other *N*-acetyl amino acids and *N*-propionyltaurine, but at lower rates (<20%) compared with *N*-acetyltaurine. No activity was observed for most of the other substrates tested, including bile salts. To determine the active-site residues important for PTER enzyme activity, we docked *N*-acetyltaurine into an AlphaFold-modelled PTER^31^. In the modelled active site, we identified residues with potential interactions with *N*-acetyltaurine (for example, H300, R233 and R204), the metal cation (for example, H26, H28 and E169), as well as other active-site residues that were proximal to the substrate (for example, Y263, Y65 and T258; Fig. 2c). A total of 15 single-point mutation bacterial recombinant mouse PTER proteins were produced and assayed for *N*-acetyltaurine hydrolysis activity in vitro (Fig. 2d). In general, the expression of these point mutants, with the exception of R233A, was comparable with that of wild-type (WT) PTER (Fig. 2d). As expected, many of these mutations completely abolished enzyme activity (Fig. 2d). We conclude that PTER is a *N*-acetyltaurine-specific hydrolase in vitro.

## Metabolite levels in *Pter* knockout mice

**Fig. 3:**
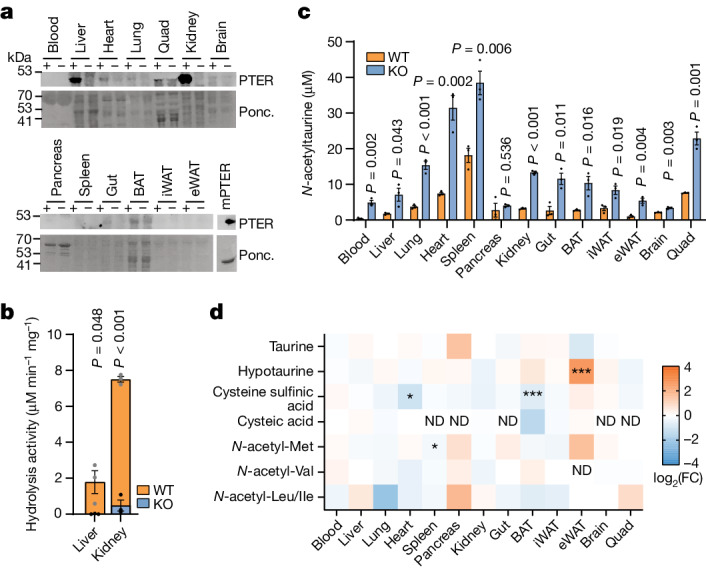
Biochemical characterization of global *Pter* KO mice. **a**, Anti-PTER blotting (top) and Ponceaus (Ponc.) staining (bottom) of the indicated total tissue lysate from 4-week-old male WT mice and *Pter* KO mice. Recombinant mouse PTER protein (mPTER; 100 ng) was used as a positive control. **b**, *N*-acetyltaurine hydrolysis activity in total lysate from the indicated tissue of WT mice and *Pter* KO mice (100 µg). Reactions were performed with 100 µM *N*-acetyltaurine for 1 h at 37 °C. *N* = 3 per group. For WT versus *Pter* KO kidney, *P* = 3.33 × 10^–5^. **c**, Absolute quantification of endogenous *N*-acetyltaurine levels in the indicated tissue from 4-week-old WT mice and *Pter* KO mice. *N* = 3 per group. For WT versus *Pter* KO lung, *P* = 6.61 × 10^–4^; for kidney, *P* = 1.12 × 10^–5^. **d**, Relative fold change (FC) of the indicated metabolites from the indicated tissue of 4-week-old WT mice and *Pter* KO mice. *N* = 3 per group. In **b–d**, data are shown as the mean ± s.e.m. For **a**, the loading control was performed on the same blot. In **b**–**d**, *P* values were calculated from two-tailed unpaired *t*-tests and not adjusted for multiple comparisons. In **d**, *P < 0.05, **P < 0.01 and ***P < 0.001. ND, not detected. Experiments were performed twice (**a**,**b**) or once (**c**,**d**). Source Data

To determine the potential physiological relevance of PTER in vivo, we obtained global *Pter* knockout (KO) mice. These animals were produced by the Knockout Mouse Phenotyping Consortium (KOMP) but had not been previously studied. Overall, *Pter* KO mice were born in the expected Mendelian ratios and were overtly normal in their home-cage behaviour. Using an anti-PTER antibody, the highest PTER protein levels were detected in liver and kidney tissues of WT mice (Fig. 3a), which corresponded to the same tissues in which we had originally detected high *N*-acetyltaurine hydrolysis activity (Fig. 1b). Complete loss of PTER protein and concomitant loss of *N*-acetyltaurine hydrolysis activity was observed in these two tissues from *Pter* KO mice (Fig. 3b). To determine whether genetic *Pter* deficiency alters endogenous *N*-acetyltaurine levels, we developed a targeted LC–MS method for this metabolite. Using this method, we observed that *Pter* KO tissues exhibited increased *N*-acetyltaurine levels, which, by magnitude, ranged from twofold (in spleen) to tenfold (in blood) (Fig. 3c). We conclude that PTER is a physiological *N*-acetyltaurine hydrolase in vivo.

Next, we used targeted LC–MS to measure tissue levels of taurine and several taurine pathway metabolites in tissues from WT mice and *Pter* KO mice. Levels of taurine itself did not exhibit any significant genotype-dependent changes in any tissue examined (Fig. 3d). Hypotaurine was increased in epididymal white adipose tissue but not in any other tissues from *Pter* KO mice. Conversely, cysteine sulfinic acid was reduced in heart and brown fat but not in other tissues in *Pter* KO mice (Fig. 3d). Finally, cysteic acid could be detected in a subset of tissues and its levels were unaltered in *Pter* KO mice (Fig. 3d).

Because PTER also exhibited modest hydrolysis activity in vitro for four additional *N*-acetyl amino acids (*N*-acetylleucine, isoleucine, methionine and valine), we used targeted metabolomics to measure the levels of these *N*-acetylated amino acids in WT mice and *Pter* KO mice. As shown in Fig. 3d, levels of *N*-acetylmethionine were largely unaltered in *Pter* KO tissues, except for a small reduction in *N*-acetylmethionine in the spleen. Levels of *N*-acetylvaline, *N*-acetylleucine and *N*-acetylisoleucine were also unchanged in *Pter* KO mice across all tissues examined. We conclude that genetic *Pter* deficiency results in broad changes in *N*-acetyltaurine levels across all tissues. Minor and tissue-specific changes in select taurine pathway metabolites and *N*-acetylmethionine were also observed.

**Extended Data Fig. 1:**
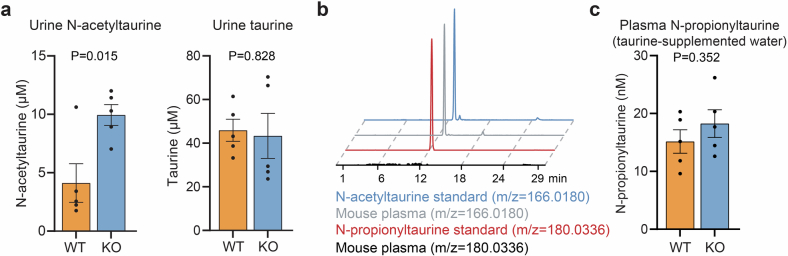
Additional characterization of metabolites in PTER-KO mice. Related to Fig. 3. **a**, Urine N-acetyltaurine concentration (left) and taurine concentration (right) of 13- to 14-week-old male WT or PTER-KO mice. N = 5/group. **b**, Representative extracted ion chromatograms of synthetic N-acetyltaurine and N-propionyltaurine standards and endogenous peaks of blood plasma from 14-week old wild-type male mice without taurine water supplementation. **c**, Plasma of N-propionyltaurine levels of 13 to 14-week-old male WT or PTER-KO mice on high fat diet and after taurine water supplementation (2.5% w/v). N = 5/group. Data are shown as mean ± SEM. In **a**, P-values were calculated from two-tailed unpaired t-tests. Source Data

In urine, *Pter* KO mice had around twofold higher *N*-acetyltaurine levels compared with WT mice, with no genotype-dependent differences in urine taurine levels (Extended Data Fig. 1a). *N*-propionyltaurine was below the limit of detection (<1 nM) in blood plasma under normal conditions. However, when mice were supplemented with taurine in drinking water (2.5% w/v), *N*-propionyltaurine was detectable at low levels (about 30 nM) and not different between WT mice and *Pter* KO mice (Extended Data Fig. 1b,c).

## Metabolic phenotypes of *Pter* KO mice

**Extended Data Fig. 2:**
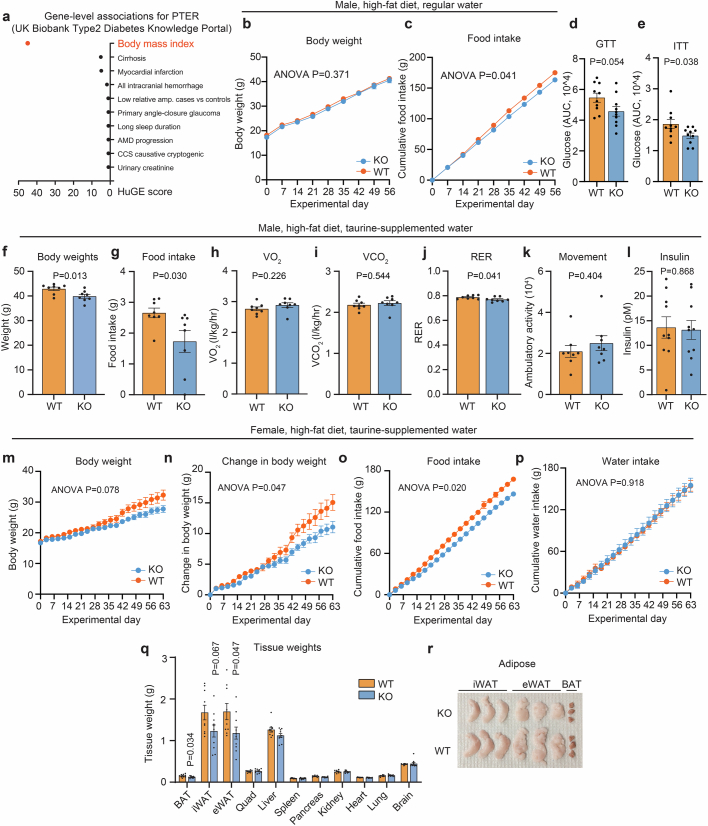
Additional metabolic characterization of male and female PTER-KO mice on high fat diet and taurine-supplemented water. Related to Fig. 4. **a**, Human genetic evidence (HuGE) score of phenotype associations for the *PTER* gene locus from the Type 2 Diabetes Knowledge Portal. **b**,**c**, Body weight (**b**) and food intake (**c**) of 12- to 13-week-old male PTER-KO mice (N = 10, blue line) or WT mice (N = 14, orange line) subjected to a high-fat diet feeding alone for a period of 8 weeks. **d**,**e**, Glucose integrated area under the curve (iAUC) for Fig. 4e,f. **f**-**k**, Metabolic chamber analysis of 12- to 13-week-old-male WT or PTER-KO mice after 8 weeks of high fat diet/taurine water supplementation (2.5% w/v). N = 8/group. RER, respiratory exchange ratio. **l**, Insulin levels of 13 to 14-week-old male WT or PTER-KO mice on high fat diet and after taurine water supplementation (2.5% w/v). N = 10/group. **m**-**r**, Body weight (**m**), change in body weight (**n**), cumulative food intake (**o**), and water intake (**p**), tissue weights (**q**) and representative adipose tissues (**r**) of 13- to 14-week-old female WT or PTER-KO mice on high fat diet and after taurine water supplementation (2.5% w/v). N = 10/group. iWAT, inguinal white adipose tissue; eWAT, epididymal white adipose tissue; BAT, brown adipose tissue; Quad, quadricep muscles. Data are shown as mean ± SEM. In **b**,**c** and **m**-**p**, P-values were calculated from two-way ANOVA with post hoc Sidak’s multiple comparisons test. In **d**-**l** and **g**, P-values were calculated from two-tailed unpaired t-tests and were not corrected for multiple comparisons. Source Data

Having established PTER as the principal *N*-acetyltaurine hydrolase in mice, we next turned to the potential functions of this biochemical pathway. A previous study^10^ identified a polymorphism near the human *PTER* gene linked to early-onset and morbid adult obesity in individuals of European ancestry^10^. Further substantiating these initial associations, in the Type 2 Diabetes Knowledge Portal, the *PTER* gene exhibited a ‘strong’ Human Genetic Evidence score linked with BMI (Extended Data Fig. 2a). These genetic data, and previous literature on the effects of taurine supplementation on energy balance and metabolism, suggest that the PTER pathway might be involved body weight regulation.

**Fig. 4:**
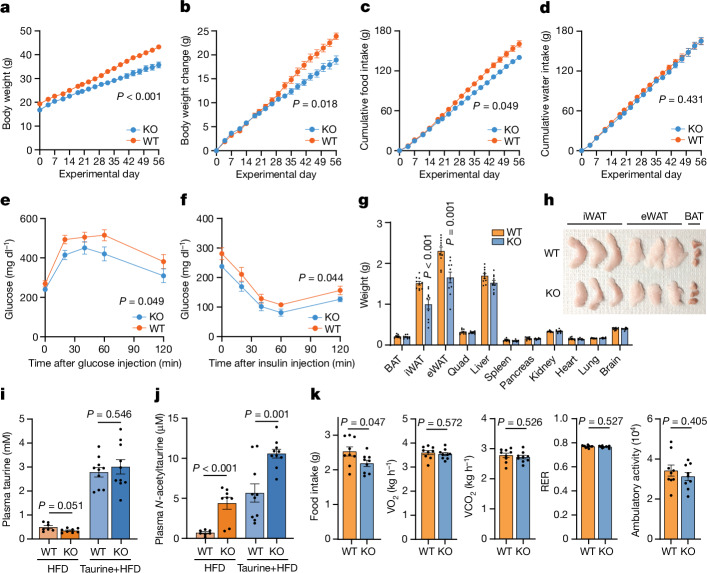
Metabolic phenotype of *Pter* KO mice. **a**–**d**, Body weight (**a**), change in body weight (**b**), cumulative food intake (**c**) and water intake (**d**) of 13–14-week-old male WT mice and *Pter* KO mice on a high-fat diet (HFD) and after taurine water supplementation (2.5% w/v). *P* = 7 × 10^–4^. *N* = 10 per group. **e**–**j**, Glucose tolerance test (**e**), insulin tolerance test (**f**), tissue weights (**g**), representative adipose tissues (**h**), plasma taurine levels (**i**) and plasma *N*-acetyltaurine levels (**j**) of 13–14-week-old male WT mice and *Pter* KO mice after 8 weeks on a high-fat diet and taurine water supplementation (2.5% w/v). For **g**, WT versus *Pter* KO iWAT, *P* = 4.97 × 10^–4^. For **j**, high-fat diet WT versus *Pter* KO blood plasma, *P* = 5.56 × 10^–4^. *N* = 10 per group. **k**, Metabolic chamber analysis of 8–9-week-old-male WT mice and *Pter* KO mice after 4 weeks of a high-fat diet and taurine water supplementation (2.5% w/v). *N* = 9 per group. RER, respiratory exchange ratio. Data are shown as the mean ± s.e.m. In **a**–**f**, *P* values were calculated from two-way analysis of variance (ANOVA) with post hoc Sidak’s multiple comparisons test. In **g**–**k**, *P* values were calculated from two-tailed unpaired *t*-tests. All experiments were performed once. Source Data

To test this prediction, we first placed a group of male *Pter* KO mice and WT littermates on a high-fat diet and monitored body weights and food intake over an 8-week period. After 8 weeks, food intake in *Pter* KO mice was reduced by a modest magnitude (about 7%), but body weight was not different (Extended Data Fig. 2b,c). Because taurine is a substrate for the PTER-catalysed reaction, we reasoned that these trends in body weight and food intake in *Pter* KO mice might be more clearly revealed under conditions when taurine flux is increased. We therefore placed new groups of WT mice and *Pter* KO mice on a high-fat diet and supplemented taurine in the drinking water (2.5% w/v). After 8 weeks under these taurine-supplemented conditions, *Pter* KO mice had lower body weight, reduced change in body weight and reduced cumulative food intake compared with WT littermates (Fig. 4a–c). Notably, water intake was equivalent between genotypes (Fig. 4d), which demonstrated that the reduced food intake in *Pter* KO mice was specific for nutrients. At the end of the experiment, *Pter* KO mice exhibited improved glucose tolerance and insulin sensitivity compared with WT mice, which probably represents a secondary effect to the lower body weight (Fig. 4e,f and Extended Data Fig. 2d,e). Dissection of tissues revealed that the difference in body weight was due entirely to a reduction in fat mass in *Pter* KO mice (Fig. 4g,h), including lower inguinal and epididymal white adipose tissue, with no changes in lean mass detected. We confirmed by MS analyses that the taurine supplementation protocol increased circulating taurine levels equivalently in both WT mice and *Pter* KO mice (Fig. 4i). Taurine supplementation in drinking water resulted in a hyperaccumulation of plasma *N*-acetyltaurine in *Pter* KO mice (Fig. 4j).

Next, we used metabolic chambers to measure parameters of whole-body energy intake and expenditure in a new group of *Pter* KO mice and WT mice given taurine-supplemented water at a time point before the divergence in body weights (4 weeks). As expected, *Pter* KO mice exhibited reduced food intake (Fig. 4k). We did not observe changes in any other measured parameters, including VO_2_, VCO_2_, respiratory exchange ratio or ambulatory movement (Fig. 4k). We then tested an independent group of male WT mice and *Pter* KO mice in metabolic chambers. Analyses at the end of the experiment after body weights had diverged (8 weeks) revealed reduced food intake and respiratory exchange ratios in *Pter* KO mice, whereas VO_2_, VCO_2_ and ambulatory movement were not different between genotypes (Extended Data Fig. 2f–k). Non-fasted insulin levels were also not different in *Pter* KO mice (Extended Data Fig. 2l).

**Extended Data Fig. 3:**
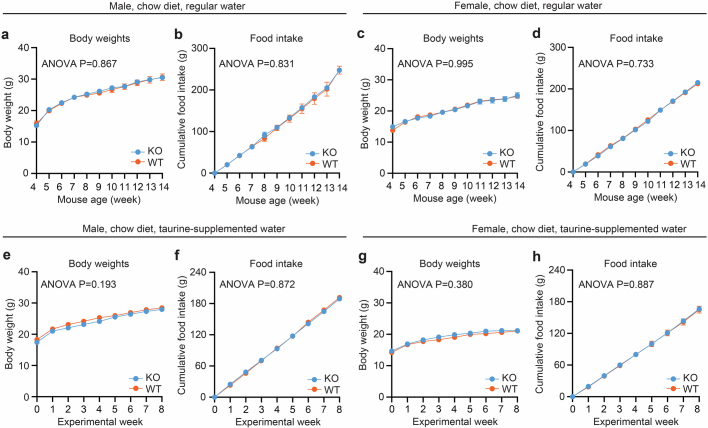
Additional metabolic characterization of chow-fed male and female PTER-KO mice. Related to Fig. 4. **a**-**h**, Body weight (**a**,**c**,**e**,**g**) and cumulative food intake (**b**,**d**,**f**,**h**) of chow-fed 12- to 14-week-old male mice (**a**,**b**,**e**,**f**) or female mice (**c**,**d**,**g**,**h**) with regular water (**a**-**d**) or with 2.5% w/v taurine-supplemented water (**e**-**h**). For **a**,**b** and **e**-**h**, N = 10/group. For **c**,**d**, N = 10 or 14/group. Data are shown as mean ± SEM. P-values were calculated from two-way ANOVA with post hoc Sidak’s multiple comparisons test. Source Data

Female *Pter* KO on the same high-fat diet and taurine-supplemented water protocol also exhibited reduced changes in body weight, food intake and adiposity compared with WT controls, without any differences in water intake (Extended Data Fig. 2m–r). The body weight and food intake phenotype, however, was absent when either male or female *Pter* KO mice were maintained on chow diet, regardless of the status of taurine supplementation in the water (Extended Data Fig. 3). We conclude that *Pter* KO mice have reduced adiposity, body weight and food intake in a stimulus-dependent manner and specifically under conditions of concurrent obesogenic diet with taurine supplementation. These data also uncover a complex gene-by-environment interaction of the *Pter* locus, taurine levels and diet.

**Extended Data Fig. 4:**
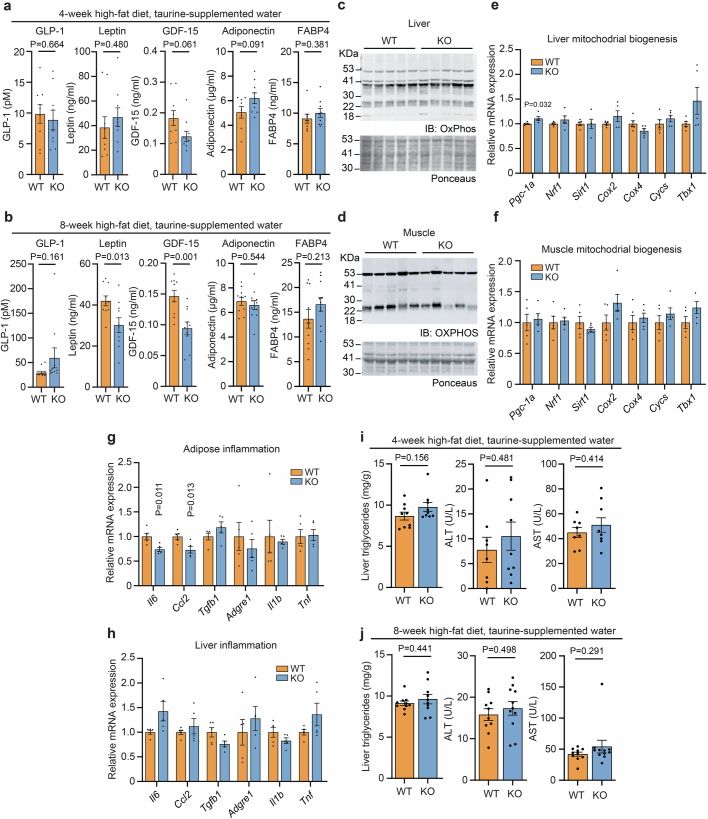
Additional molecular characterization of PTER-KO mice. Related to Fig. 4. **a**,**b**, Blood plasma levels of the indicated hormones from 8 to 9-week-old-male WT or PTER-KO mice after 4 weeks of high fat diet/taurine water supplementation (2.5% w/v) (**a**) or from 12- to 13-week-old-male WT or PTER-KO mice after 8 weeks of high fat diet/taurine water supplementation (2.5% w/v) (**b**). In **a**, N = 8/group. In **b**, N = 10/group. **c**,**d**, Western blotting with anti-OXPHOS cocktail antibody (top) and Ponceaus stain (bottom) of liver (**c**) and quadricep muscles (**d**) from 12- to 13-week-old-male WT or PTER-KO mice after 8 weeks of high fat diet/taurine water supplementation (2.5% w/v). **e**-**h**, mRNA expression of indicated genes in liver (**e**,**h**), quadricep muscles (**f**), or epididymal white adipose tissue (**g**) from 12 to 13-week-old-male WT or PTER-KO mice after 8 weeks of high fat diet/taurine water supplementation (2.5% w/v). N = 5/group. **i**,**j**, Liver triglycerides and liver enzymes from 8- to 9-week-old-male WT or PTER-KO mice after 4 weeks of high fat diet/taurine water supplementation (2.5% w/v) (**l**) or 12- to 13-week-old-male WT or PTER-KO mice after 8 weeks of high fat diet/taurine water supplementation (2.5% w/v) (**j**). In **i**, N = 8/group. In **j**, N = 10/group. Data are shown as mean ± SEM. For **c**,**d**, the loading controls were performed on the same blot. P-values were calculated from two-tailed unpaired t-tests and were not corrected for multiple comparisons. Source Data

In *Pter* KO mice and WT mice on a high-fat diet and taurine in water, no differences in levels of non-fasting plasma GLP-1, leptin, GDF15, ghrelin, FABP4 or adiponectin were observed at the 4-week time point (Extended Data Fig. 4a). At the 8-week time point, plasma leptin and plasma GDF15 levels were reduced in *Pter* KO mice (Extended Data Fig. 4b), a result consistent with the reduced adiposity and obesity of these animals at that time point. Protein levels of mitochondrial complexes in either liver or muscle tissue were not different between *Pter* KO mice and WT mice at the 8-week time point (Extended Data Fig. 4c,d). Similarly, mRNA levels of mitochondrial markers or mitochondrial biogenesis markers were not different between genotypes in these two tissues (Extended Data Fig. 4e,f). At the 8-week time point, mRNA levels of the cytokines *Il1* and *Ccl2* were modestly reduced in adipose tissues from *Pter* KO mice (Extended Data Fig. 4g), whereas mRNA levels of these cytokines were not different in liver (Extended Data Fig. 4h). Liver triglycerides, AST and ALT levels were also not different between genotypes (Extended Data Fig. 4i,j).

**Extended Data Fig. 5:**
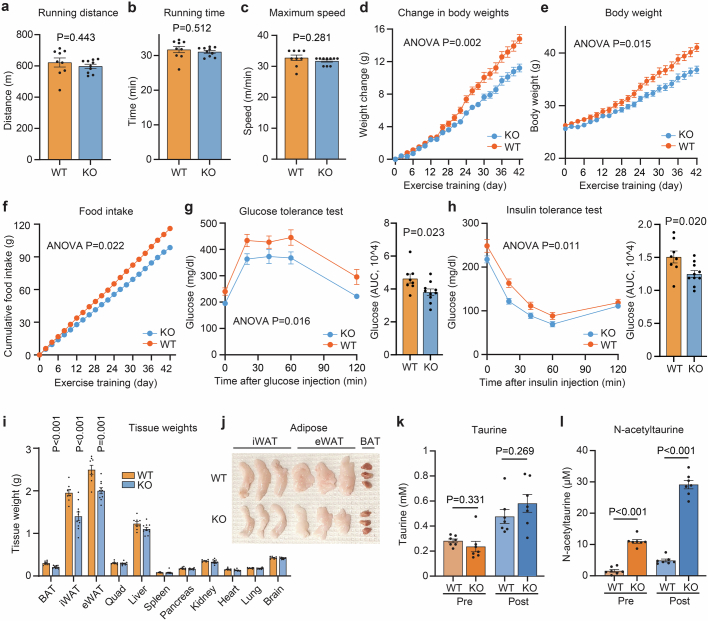
Metabolic phenotype of male PTER-KO mice on a chronic treadmill exercise training protocol. Related to Fig. 4. **a**-**c**, Running distance (**a**), running time (**b**) and maximum speed (**c**) of 10- to 11-week-old male PTER-KO mice (N = 10, blue line and box) or WT mice (N = 9, orange line and box) following a single bout of treadmill exercise running until exhaustion. **d**-**l**, Change in body weight (**d**), body weight (**e**), cumulative food intake (**f**), glucose tolerance test (**g**), insulin tolerance test (**h**), tissues weights (**i**), representative adipose tissues (**j**), plasma taurine levels (**k**), and plasma N-acetyltaurine levels (**l**) of 10- to 11-week-old male PTER-KO mice (N = 10, blue line and box) or WT mice (N = 8, orange line and box) after a 6-week treadmill exercise training protocol (see Methods). For **i**, WT versus PTER-KO BAT, P = 1.41e-04; iWAT, P = 6.98e-04, eWAT, P = 103e-03. For **l**, WT versus PTER-KO pre-exercise, P = 2.94e-08; post-exercise, P = 8.98e-10. Data are shown as mean ± SEM. In **a**-**c** and **i**-**l**, P-values were calculated from two-tailed unpaired t-tests and were not corrected for multiple comparisons. In **d**-**h**, P-values were calculated from two-way ANOVA with post hoc Sidak’s multiple comparisons test. Source Data

As an independent test of the stimulus-dependent body weight phenotype in *Pter* KO mice, we subjected a new group of male WT mice and *Pter* KO mice to a combined high-fat diet and treadmill-running protocol (Extended Data Fig. 5). We selected treadmill exercise as a second physiological stimulus because of its previously reported effects of increasing taurine and *N*-acetyltaurine levels^7,19^ (Methods). We did not observe any differences in running speed or distance in WT mice and *Pter* KO mice (Extended Data Fig. 5a–c). *Pter* KO mice again gained less weight and had reduced food intake compared with WT mice (Extended Data Fig. 5d–f). *Pter* KO mice subjected to treadmill exercise also exhibited improved glucose tolerance and insulin sensitivity compared with WT mice (Extended Data Fig. 5g,h). Dissection of tissues revealed that the weight difference was once again largely due to reductions in adipose tissue mass (Extended Data Fig. 5i,j). Last, under this the high-fat diet and treadmill-running protocol, we confirmed that taurine levels increased by around twofold in both WT mice and *Pter* KO mice (Extended Data Fig. 5k). Similarly, plasma *N*-acetyltaurine levels in *Pter* KO mice subjected to exercise reached a level much higher than that of WT mice (with or without exercise) or even sedentary *Pter* KO mice (Extended Data Fig. 5l).

## Metabolic effects of *N*-acetyltaurine

**Fig. 5:**
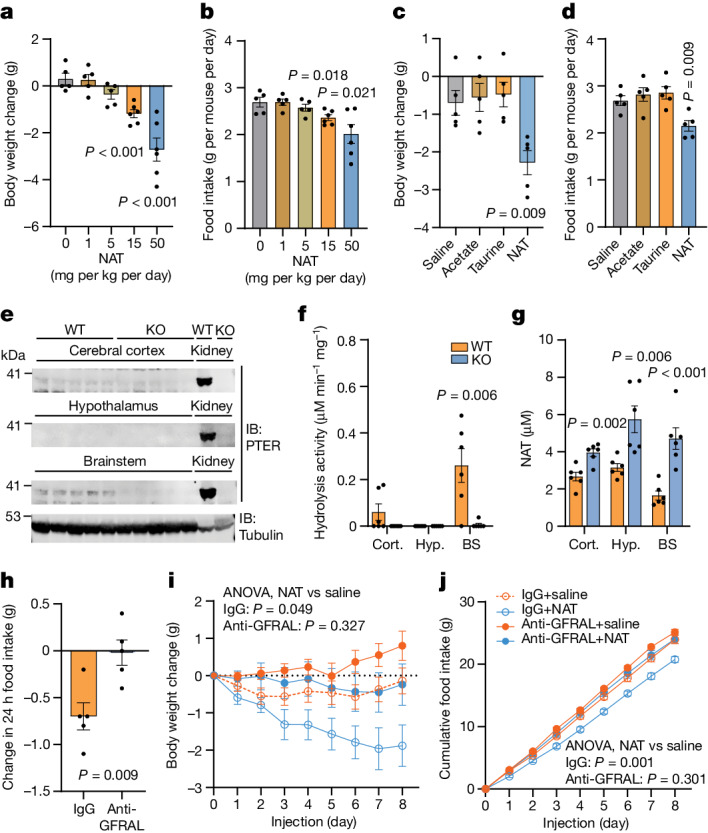
Effect of *N*-acetyltaurine administration in mice. **a**,**b**, Change in body weight (**a**) and food intake (**b**) of 26–28-week-old male DIO C57BL/6J mice following 7 days of treatment with the indicated dose of *N*-acetyltaurine (NAT; i.p.). For saline versus *N*-acetyltaurine (15 mg per kg per day), *P* = 5.95 × 10^–4^; for saline versus *N*-acetyltaurine (50 mg per kg per day), *P* = 6.3 × 10^–4^. *N* = 5 per group for vehicle, 1 and 5 mg per kg per day; *N* = 6 per group for 15 and 50 mg per kg per day. **c**,**d**, Change in body weight (**c**) and food intake (**d**) of 19–21-week-old male DIO C57BL/6J mice following treatment with the indicated metabolite at a dose of 15 mg per kg per day (i.p.). *N* = 5 per group. **e**–**g**, Western blots with anti-PTER (top) and anti-tubulin (bottom) antibodies (**e**), *N*-acetyltaurine hydrolysis activity (**f**) and tissue *N*-acetyltaurine levels (**g**) from cortex (Cort.), hypothalamus (Hyp.) and brainstem (BS) of WT mice and *Pter* KO mice. For WT versus *Pter* KO brainstem, *P* = 6.65 × 10^–4^. *N* = 6 per group for **f** and **g**. **h**, Change in 24-h food intake of 6-month-old male DIO mice treated with a single dose of GDF15 (0.1 mg kg^–1^, i.p.) in the presence of anti-GFRAL antibody (10 mg kg^–1^, i.p.) or IgG control antibody (10 mg kg^–1^, i.p.). *N* = 5 per group. **i**,**j**, Change in body weight (**i**) and cumulative food intake (**j**) of 16-week-old male DIO mice following saline or *N*-acetyltaurine (15 mg per kg per day, i.p.) treatment and with IgG or anti-GFRAL antibody co-treatment (10 mg kg^–1^, i.p., once every 3 days). *N* = 10 per group. Data are shown as the mean ± s.e.m. For **e**, the loading control was performed on the same blot. In **a**–**d** and **f**–**h**, *P* values were calculated from two-tailed unpaired *t*-tests and were not corrected for multiple comparisons. In **i** and **j**, *P* values were calculated from two-way ANOVA with post hoc Sidak’s multiple comparisons test. All experiments were performed once. Source Data

**Extended Data Fig. 6:**
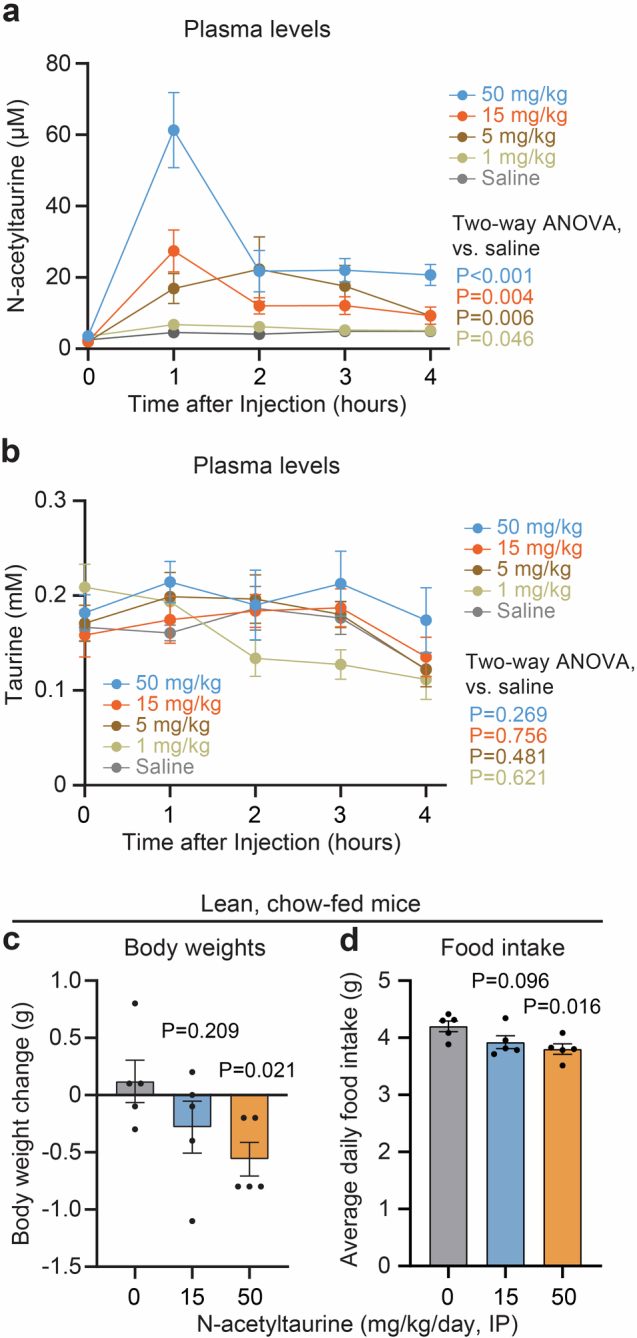
Additional characterization of N-acetyltaurine administration to wild-type mice. Related to Fig. 5. **a**,**b**, Blood plasma concentrations of N-acetyltaurine (**a**) or taurine (**b**) from 26- to 28-week-old male DIO C57BL/6J mice after intraperitoneal injection with indicated doses of N-acetyltaurine. For saline versus N-acetyltaurine (50 mg/kg), P = 2e-04. N = 5/group. **c**,**d**, Body weight change (**c**) and food intake (**d**) of 13-week-old chow-fed male C57BL/6J mice following 7 days of treatment with the indicated dose of N-acetyltaurine (intraperitoneal injection). N = 5/group. Data are shown as mean ± SEM. In **c**,**d**, P-values were calculated from two-tailed unpaired t-tests. In **a**,**b**, P-values were calculated from two-way ANOVA with post hoc Sidak’s multiple comparisons test. Source Data

Because accumulation of *N*-acetyltaurine was the major metabolite difference between WT mice and *Pter* KO mice, we sought to determine whether *N*-acetyltaurine administration alone is sufficient to reproduce aspects of the energy balance phenotype of *Pter* KO mice. We administered *N*-acetyltaurine to diet-induced obese (DIO) mice (1–50 mg per kg per day, intraperitoneally (i.p.)). After a single administration of *N*-acetyltaurine, we observed large increases in plasma *N*-acetyltaurine levels that at one hour reached a concentration of about 30 and 60 μM at the 15 and 50 mg kg^–1^ (i.p.) dose, respectively (Extended Data Fig. 6a), without any changes to plasma taurine levels (Extended Data Fig. 6b). After chronic daily dosing, DIO mice treated with *N*-acetyltaurine exhibited dose-dependent reductions in both body weight (Fig. 5a) and food intake (Fig. 5b). In lean mice, *N*-acetyltaurine also suppressed food intake and body weight, but with a magnitude that was reduced compared with the effect observed in DIO mice (Extended Data Fig. 6c,d). To determine whether the effect of *N*-acetyltaurine required the intact amidated conjugate, we performed head-to-head comparisons of the effects of *N*-acetyltaurine with either acetate alone or taurine alone all at the same dose (15 mg per kg per day). Mice treated with *N*-acetyltaurine exhibited reduced food intake and body weight, whereas mice treated with either acetate alone or taurine alone were indistinguishable from vehicle-treated mice (Fig. 5c,d). We conclude that administration of *N*-acetyltaurine to WT mice is sufficient to reduce body weight and food intake.

**Extended Data Fig. 7:**
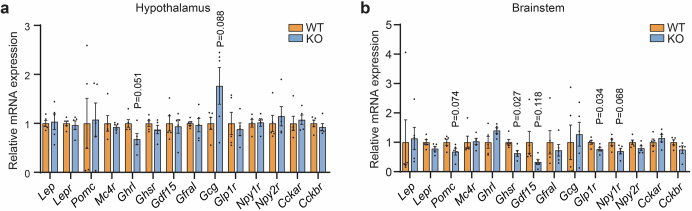
Gene expression in hypothalamus and brainstem from PTER-KO mice. Related to Fig. 5. **a**,**b**, mRNA expression of indicated genes of hypothalamus (**a**) and brainstem (**b**) from 12- to 13-week-old-male WT or PTER-KO mice after 8 weeks of high fat diet/taurine water supplementation (2.5% w/v). N = 5/group. Data are shown as mean ± SEM. P-values were calculated from two-tailed unpaired t-tests. Source Data

To better understand how *N*-acetyltaurine controls feeding behaviours, we examined the expression of PTER in various brain regions by western blotting using our anti-PTER antibody. PTER was detected in the brainstem but not hypothalamus or cerebral cortex (Fig. 5e). We also detected PTER-dependent hydrolysis activity in the brainstem and accumulation of *N*-acetyltaurine in that region (Fig. 5f,g). Although *N*-acetyltaurine was increased in all the brain regions examined, the greatest fold change was observed in the brainstem (Fig. 5h). Profiling of mRNA for neuropeptide and feeding-related genes in both the brainstem and the hypothalamus did not reveal any obvious PTER-dependent changes of large magnitude (Extended Data Fig. 7).

**Extended Data Fig. 8:**
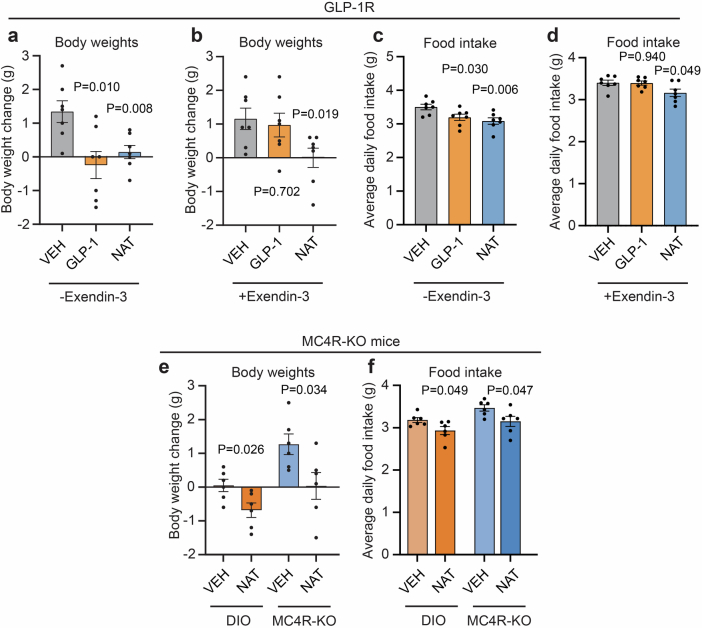
Requirement for GLP1R and MC4R in the anorexigenic effects of N-acetyltaurine. Related to Fig. 5. **a**-**d**, Body weight (**a**,**b**) and food intake (**c**,**d**) of 6 to 7-month-old DIO male C57BL/6J mice after a 7-day treatment of saline or N-acetyltaurine (15 mg/kg/day, IP) or GLP-1 (2 mg/kg/day, IP) with or without Exendin-3 (0.1 mg/kg/day, IP). N = 7/group. NAT, N-acetyltaurine. **e**,**f**, Body weight (**e**) and food intake (**f**) of 5-month-old DIO male C57BL/6J mice or 3 to 4-month-old MC4R-KO mice on high fat diet after a 7-day treatment of saline or N-acetyltaurine (15 mg/kg/day, IP). N = 6/group. NAT, N-acetyltaurine. **g**, Plasma GDF15 (left), GLP-1 (middle) and leptin (right) levels of 19 to 21-week-old male DIO C57BL/6J mice following treatment with N-acetyltaurine 15 mg/kg/day (IP) or saline. N = 5 per group. NAT, N-acetyltaurine. Data are shown as mean ± SEM. P-values were calculated from two-tailed unpaired t-tests. Source Data

Because of the established role of brainstem-restricted GDF15–GFRAL signalling in feeding control, we tested whether the anorexigenic and anti-obesity effects of *N*-acetyltaurine administration requires an intact GFRAL receptor. We obtained a neutralizing anti-GFRAL antibody (IgG clone 8A2) and an IgG control antibody. We confirmed that the anti-GFRAL antibody abrogated the anorexigenic effect of recombinant GDF15 (Fig. 5i). As expected, *N*-acetyltaurine reduced body weight and food intake when co-administered with the IgG control antibody (Fig. 5j,k). By contrast, *N*-acetyltaurine did not significantly reduce either body weight or food intake in the presence of anti-GFRAL antibody (Fig. 5j,k). We also tested the role of GLP-1R and hypothalamic MC4R signalling in the anti-obesity effects of *N*-acetyltaurine. The GLP-1R antagonist exendin-3 blocked the effects of GLP-1 peptide in food intake and body weight. However, under these conditions, exendin-3 did not blunt the body-weight-lowering effect of *N*-acetyltaurine (Extended Data Fig. 8a–d). Similarly, *N*-acetyltaurine suppressed food intake and body weight in *Mc4r* KO mice (Extended Data Fig. 8e,f). Last, administration of *N*-acetyltaurine to mice increased circulating GDF15 levels by about 40%, whereas leptin and GLP-1 levels remained unchanged (Extended Data Fig. 8g). We conclude that PTER is expressed in the brainstem and that the full anorexigenic and anti-obesity effects of *N*-acetyltaurine require functional GFRAL receptors.

**Extended Data Fig. 9:**
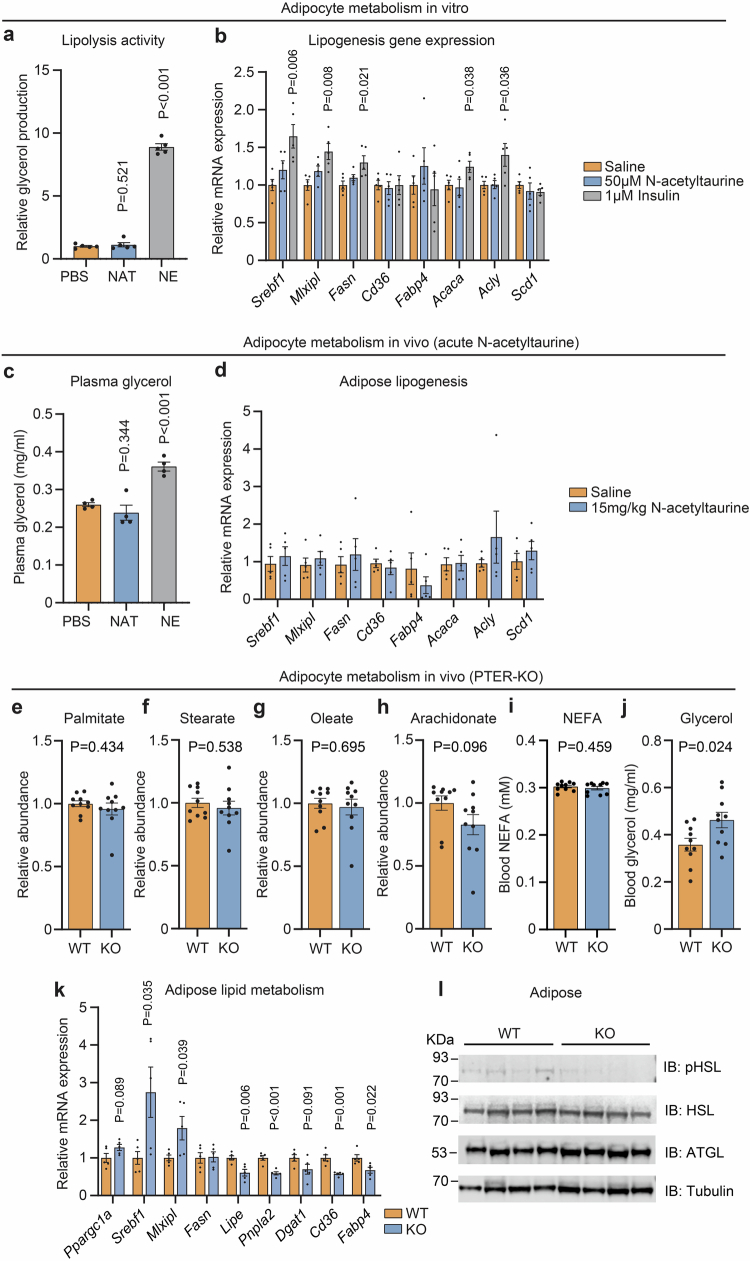
Direct and indirect effects of N-acetyltaurine on adipose. Related to Fig. 5. **a**, Glycerol production from mature epididymal adipocytes isolated from 4-month-old male C57BL/6J mice after incubation with 50 μM N-acetyltaurine (NAT) or 1 μM norepinephrine (NE) at 37 °C for 1 h. For PBS versus NE, P = 1.72e-09. N = 5/group. **b**, mRNA expression of indicated genes from mature epididymal adipocytes isolated from 4-month-old male C57BL/6J mice and incubated with PBS or 50 μM N-acetyltaurine or 1 μM insulin at 37 °C for 4 h with constant shaking. N = 5/group. **c**, Plasma glycerol levels of 4-month-old male DIO C57BL/6J mice one hour after a single administration of N-acetyltaurine (NAT, 15 mg/kg, IP) or norepinephrine (NE, 0.5 mg/kg, IP) treatment. For PBS versus NE, P = 2.33e-04. N = 4/group. **d**, mRNA expression of indicated genes from epididymal white adipose tissues of 4-month-old male DIO C57BL/6J mice four hours after a single administration of N-acetyltaurine (15 mg/kg, IP) treatment. N = 5/group. **e**-**j**, Plasma palmitate (**e**), stearate (**f**), oleate (**g**), arachidonate (**h**), total non-esterified fatty acids (**i**), and glycerol (**j**), mRNA expression of indicated genes from epididymal white adipose tissue (**k**), Western blotting of epididymal white adipose tissues with the indicated antibodies (**l**) of 13 to 14-week-old male WT or PTER-KO mice after 8 weeks on high fat diet and taurine water supplementation (2.5% w/v). For WT versus PTER-KO *Pnpla2*, P = 6.03e-04. In **e**-**i**, N = 10/group. In **k**, N = 5/group. In **l**, N = 4/group. Data are shown as mean ± SEM. For **l**, the loading control was performed on the same blot. In **a**-**j**, P-values were calculated from two-tailed unpaired t-tests and were not corrected for multiple comparisons. Source Data

To determine the direct versus indirect effects of *N*-acetyltaurine in adipose tissues, we examined the effects of *N*-acetyltaurine in isolated adipocytes in vitro and after administration to mice in vivo. In vitro, *N*-acetyltaurine did not acutely stimulate adipocyte lipolysis, as measured by glycerol release (Extended Data Fig. 9a). *N*-acetyltaurine also did not stimulate the expression of lipogenesis or lipid uptake-associated genes in isolated adipocytes (Extended Data Fig. 9b). A single administration of *N*-acetyltaurine to mice did not stimulate lipolysis or alter lipogenesis or lipid-uptake gene expression in epididymal fat tissues (Extended Data Fig. 9c,d). Therefore, *N*-acetyltaurine does not directly regulate lipid metabolism in adipocytes. In plasma from *Pter* KO mice, we did not observe any changes in specific or total plasma free-fatty acid species, whereas plasma glycerol levels were modestly increased (Extended Data Fig. 9e–j). In epididymal fat from *Pter* KO mice, complex bidirectional changes in mRNA levels for lipid uptake and lipogenesis genes were observed, and phosphorylation of hormone-sensitive lipase (HSL) was slightly reduced (Extended Data Fig. 9k,l), all of which probably represent secondary effects due to reduced food intake in *N*-acetyltaurine-treated mice.

## Pathways of *N*-acetyltaurine production

**Extended Data Fig. 10:**
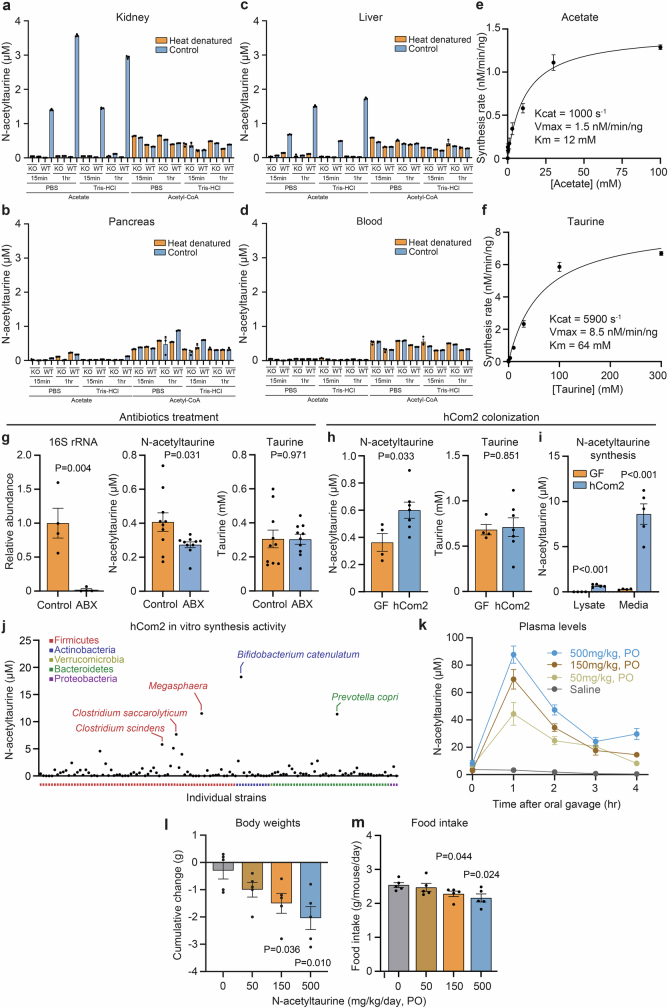
Metabolic pathways for N-acetyltaurine production. Related to Fig. 5. **a**-**d**, Production of N-acetyltaurine from cell lysates of kidney (**a**), pancreas (**b**), liver (**c**), or blood plasma (100 µg) following incubation at 37 °C under the indicated buffer condition and incubation time. The following concentrations of substrates were used: taurine (10 mM), acetate (10 mM), acetyl-CoA (100 µM). N = 3/condition. **e**,**f**, Rate of N-acetyltaurine production following incubation of purified recombinant mPTER with the indicated concentration of acetate and 10 mM taurine (**e**) or the indicated concentration of taurine and 10 mM acetate (**f**) at at 37 °C for 1 hr. N = 3/group. **g**, 16S rRNA levels (left), plasma N-acetyltaurine (middle), and plasma taurine (right) levels of control and antibiotic (ABX)-treated 12- to 14-week-old wild-type lean male mice. Antibiotic mixture (chloramphenicol, spectinomycin dihydrochloride pentahydrate, apramycin sulfate, tetracycline hydrochloride, kanamycin and ampicillin at 1 g/l per antibotic) was administered in drinking water ad libitum and orally gavaged (0.5 ml) every other day for a whole duration of 2 weeks. N = 10/group. **h**, Plasma N-acetyltaurine and plasma taurine levels of germ-free (GF) or hCom2-colonized 12- to 14-week-old wild-type lean male mice. N = 4 for GF mice and N = 4 for hCom2-colonized mice. **i**, N-acetyltaurine levels in cell lysates or media of cells isolated from feces of GF or hCom2-colonized 12- to 14-week-old wild-type lean male mice. For GF versus hCom2 cell lysate; P = 9.45e-05; medium, P = 3.43e-04. N = 4 for GF mice and N = 5 for hCom2-colonzied mice. **j**, Media N-acetyltaurine levels following incubation of taurine (10 mM) and acetate (10 mM) for 48 h with individual bacterial strains from hCom2. N = 2/strain. **k**, Blood plasma N-acetyltaurine levels from 7-month-old male DIO C57BL/6J mice after oral gavage with indicated doses of N-acetyltaurine. N = 5/group. **l**,**m**, Change in body weight (**l**) and food intake (**m**) of 3-month-old male DIO mice treated orally with the indicated dose of N-acetyltaurine over a 7-day period (50–500 mg/kg/day, PO). N = 5/group. Data are shown as mean ± SEM. In **g**-**i** and **l**,**m**, P-values were calculated from two-tailed unpaired t-tests. Data in **e**,**f** were fitted to Michaelis–Menten kinetics (solid line) using GraphPad Prism. Source Data

Last, we sought to understand the potential pathways of *N*-acetyltaurine biosynthesis from taurine. To determine whether taurine *N*-acetyltransferase activity could be detected in mouse tissues, we measured *N*-acetyltaurine production in liver, kidney, brain and blood plasma from WT mice and *Pter* KO mice using various incubation times, buffers and acetyl donors (acetate and acetyl-CoA) with taurine. Notably, we observed PTER-dependent *N*-acetyltaurine biosynthesis activity using taurine and acetate as substrates (Extended Data Fig. 10a–d). Recombinant PTER catalysed taurine *N*-acetyltransferase in vitro (*K*_m_ = 65 mM for taurine and *K*_m_ = 12 mM for acetate; Extended Data Fig. 10e,f). Therefore PTER can operate in ‘reverse’ when provided high concentrations of acetate and taurine as substrates. In addition, we observed non-enzymatic production of *N*-acetyltaurine through condensation of acetyl-CoA with taurine (Extended Data Fig. 10a–d). We also considered the possibility that the gut microbiome may be a source of *N*-acetyltaurine. Indeed, WT mice treated with an antibiotic cocktail for 1 week exhibited an approximately 30% reduction in circulating *N*-acetyltaurine levels without any changes in circulating taurine levels (Extended Data Fig. 10g). Conversely, plasma *N*-acetyltaurine, but not taurine, was increased by about 80% after colonization of germ-free mice with the defined microbial community hCom2 (ref. ^32^) (Extended Data Fig. 10h). Using a biochemical assay to measure taurine *N*-acetyltransferase activity, we detected strong production and secretion of *N*-acetyltaurine in the cellular fraction of faeces isolated from hCOM2-colonized mice but not in germ-free mice (Extended Data Fig. 10i). Consistent with these in vivo data, individual strains of hCom2 also exhibited *N*-acetyltaurine production activity in vitro (Extended Data Fig. 10j). Oral administration of *N*-acetyltaurine to DIO mice dose-dependently increased plasma *N*-acetyltaurine levels and reduced food intake and body weight (Extended Data Fig. 10k–m), which demonstrated that *N*-acetyltaurine can cross the intestinal barrier intact. These data provide evidence for at least three distinct pathways that can contribute to *N*-acetyltaurine production in vivo: reverse PTER-mediated synthesis, non-enzymatic taurine condensation with acetyl-CoA and the microbiome.

## Discussion

Until now, *N*-acetyltaurine has been a largely understudied secondary taurine metabolite. In addition, PTER has been an enigmatic enzyme that had remained largely uncharacterized with respect to both biochemical activity and physiological function. Here we showed that PTER catalyses the hydrolysis of *N*-acetyltaurine into taurine and acetate, a reaction that places PTER into a previously unrecognized and central enzymatic node in taurine metabolism. We also demonstrated that genetic loss of *Pter* or pharmacological administration of *N*-acetyltaurine results in reduced food intake, adiposity and body weight under obesogenic conditions with a taurine flux stimulus. Notably, obesity alone, at least in mice, showed a subtle PTER-dependent effect, which underscored the complex gene-by-environment interaction of the *Pter* locus, taurine levels and diet. These data also suggest that the mechanistic basis for the genetic associations between the *PTER* locus and BMI in humans may involve *N*-acetyltaurine. In the future, the development of potent and selective PTER inhibitors may enable pharmacological targeting of this biochemical pathway for the treatment of obesity.

A major unanswered question is the precise molecular and circuit mechanisms by which PTER and *N*-acetyltaurine regulate feeding behaviours and energy balance. Our data point to the brainstem and GFRAL receptors as contributors to the downstream effects of *N*-acetyltaurine. Because increased GDF15–GFRAL signalling is associated with nausea, these data also suggest that nausea may be driving, at least in part, the reduction in food intake after pharmacologically mediated increases in *N*-acetyltaurine. Whether GFRAL and nausea is an important component of the *Pter* KO phenotype would require additional studies with the neutralizing anti-GFRAL antibody in *Pter* KO mice. We suspect that *N*-acetyltaurine probably does not directly bind GFRAL itself because *N*-acetyltaurine is a metabolite and consequently does not share any structural similarity with the natural GFRAL ligand GDF15. The specific pathway of the crosstalk between *N*-acetyltaurine and GFRAL may be complex and involve intermediate steps. For example, *N*-acetyltaurine may modulate neurotransmission pathways upstream of GFRAL. Indeed, *N*-acetyltaurine shares structural similarity with the neurotransmitter acetylcholine, and taurine itself has been shown to be an agonist of GABA_A_ and glycine receptors^2^. Conditional *Pter* alleles, which are currently being developed in our laboratory, will enable dissection of the central versus peripheral contributions of PTER to the whole-body energy balance phenotypes. Last, although acute *N*-acetyltaurine administration did not have any effects in adipose tissues in vitro or in vivo, in the future, more thorough evaluation of chronic *N*-acetyltaurine treatment on adipose function is warranted.

A second open question relates to the metabolic pathways responsible for *N*-acetyltaurine biosynthesis. We provide evidence for at least three possibilities: the reverse PTER reaction, non-enzymatic condensation of acetyl-CoA with taurine and gut microbiome-dependent *N*-acetyltaurine production. Additional pathways involved in the biosynthesis and/or metabolism of *N*-acetyltaurine might be revealed by a more careful survey of in vitro enzyme assay conditions beyond those tested here. In addition, turnover flux measurements of taurine and *N*-acetyltaurine, especially under diverse physiological stimuli, and in WT mice and *Pter* KO mice would be valuable to increase our understanding of the kinetics and dynamic regulation of these metabolites in vivo. In the future, it will be important to continue to develop an understanding of the physiological contexts and regulators of *N*-acetyltaurine production; such knowledge will be essential to inform our understanding of the logic and teleology of the *N*-acetyltaurine pathway in energy balance. That *N*-acetyltaurine is under co-regulation by both host and microbial pathways also raises the possibility that rational manipulation of the gut microbiome may be a viable strategy for augmenting host *N*-acetyltaurine levels to reduce body weight.

In recent years, there has been an increased interest in taurine and taurine supplementation for many other aspects of human health and disease beyond obesity and metabolism. For instance, taurine has been linked to multiple age-associated phenotypes^19,33,34^. Our data show that that secondary taurine metabolites, such as *N*-acetyltaurine, are not simply biomarkers or inert by-products but in fact chemical effectors of the increased taurine state. Future studies that explore the role of *N*-acetyltaurine in these other processes may identify opportunities in which pharmacological manipulation of secondary taurine metabolism may be therapeutically useful.

## Methods

### Chemicals

dl-Dithiothreitol (DTT) (D0632-1G), taurine (T0625-100G), acetate (S2889-250G), isoleucine (I2752-1G), l-methionine (M5308-25G), l-leucine (L8000-25G), l-valine (V-0500), l-serine (S260-0), l-proline (P0380-100G), l-threonine (T8625-1G), l-alanine (A7627-1G), β-alanine (05160-50G), l-arginine (A5006-100G), l-cysteine (168149-25G), l-glutamic acid (49621-250G), l-glutamine (G-3126), l-histidine (H-8000), l-tryptophan (T0254-5G), l-asparagine (A0884-25G), l-lysine (L5501-5G), acetate (S2889-250G), propionate (P1880-100G), butyrate (B5887-1G), palmitate (P9767-5G), oleate (O7501-1G), stearate (S3381-5G), arachidonate (10931), *N*-acetyl-l-methionine (01310-5G), *N*-acetyl-l-leucine (441511-25G), *N*-acetyl-l-phenylalanine (857459-5G), *N*-acetyl-l-tyrosine (PHR1173-1G), *N*-acetyl-l-serine (A2638-1G), *N*-acetyl-l-proline (A0783-1G), *N*-acetyl-l-alanine (A4625-1G), *N*-acetyl-l-arginine (A3133-5G), *N*-acetyl-l-cysteine (A7250-25G), *N*-acetyl-l-glutamic acid (855642-25G), *N*-acetyl-l-glutamine (A9125-25G), *N*-acetyl-l-tryptophan (A6376-10G), *N*-acetyl-glycine (A16300-5G), *N*-acetyl-l-asparagine (441554-1G), *N*-acetyl-l-lysine (A2010-1G), *N*-acetyl-l-aspartic acid (00920-5G), chloramphenicol (C0378-25G), spectinomycin dihydrochloride pentahydrate (S4014-25G), apramycin sulfate (A2024-5G), tetracycline hydrochloride (T7660-5G) and ampicillin (A9518) were purchased from Sigma. Paraformaldehyde (AAJ19943K2), tryptone (BP1421-500), yeast extract (BP1422-500), l-tyrosine (A11141.22), glycine (G48-212), *N*-acetyl-β-alanine (H50208.03) and kanamycin (11815032) were purchased from Thermo Scientific. *N*-acetyltaurine (35169), lithocholate (20253), α-muricholate (20291), taurocholate (16215), *N*-palmitoyl-taurine (10005611), *N*-oleoyl-taurine (10005609), *N*-stearoyl-taurine (10005610), *N*-arachidonoyl-taurine (10005537), taurolithocholic acid (17275), tauro-α-muricholic acid (20288) and taurocholic acid (16215) were purchased from Cayman. l-Phenylalanine (A13238), *N*-*N*-acetyl-l-isoleucine (H66771), *N*-acetyl-l-valine (H66943) and *N*-acetyl-l-histidine (J65657) were purchased from Alfa Aesar. l-Aspartic acid (11625) was purchased from United States Biochemical. *N*-acetyl-l-threonine (03262) was purchased from Chem-Impex Int’l. Heavy *N*-acetyltaurine and *N*-propionyl-taurine were synthesized by Acme. GLP-1 (7–37) peptides (CP0005) were purchased from Genescript. Exendin-3 (9–39) amide (2081) was purchased from Tocris. Recombinant GDF15 (957-GD) was purchased from R&D Systems. Anti-GFRAL neutralizing antibody and control IgG antibody^35^ were obtained from Eli Lilly, a gift provided by P. Emmerson.

### Cell line culture

The HEK293T cell line was obtained from the American Type Culture Collection (ATCC) and grown at 37 °C with 5% CO_2_. The culture medium consisted of Dulbecco’s modified Eagle’s medium (Corning, 10-017-CV) with 10% FBS (Corning, 35010CV) and 1:1,000 penicillin–streptomycin (Gibco, 15140-122). For transient transfection, cells were transfected in 10 cm^2^ at about 60% confluency using PolyFect (Qiagen, 301107) and washed with complete culture medium 6 h later. The HEK293T cells were negative following testing for mycoplasma contamination.

### Generation of *PTER* KO cells

The pLentiCRISPRv2 system was used to generate *PTER* KO HEK293T cells. The single guide RNA (sgRNA) used was 5′-GATGGAACCAGTATCAAGTG-3′. The following oligonucleotides were used to clone the sgRNA into the plentiCRISPRv2 vector: forward, 5′-CACCGGATGGAACCAGTATCAAGTG-3′; reverse, 5′-AAACCACTTGATACTGGTTCCATCC-3′. Lentiviral particles were produced in the HEK293T cell line using PolyFect for the co-transfection of the cloned plentiCRISPRv2 plasmid with the viral packing psPAX2 plasmid and the viral envelope pMD2.G plasmid. A plentiCRISPRv2 plasmid without any sgRNA insert was used as a negative control. Medium containing lentivirus was collected 48 h after transfection and filtered through a 0.45-µm filter. The supernatant was then mixed in a 1:1 ratio with polybrene (Sigma, TR-1003-G) to a final concentration of 8 µg ml^–1^ polybrene. The viral mixture was added to HEK293T cells at 40–50% confluence in 6-well plates. Transduced cells were transferred to a 10 cm^2^ plate and subjected to puromycin selection for a period of 3–6 days. Surviving cells were then trypsinized, resuspended and plated at a 10,000× dilution to a new 10 cm^2^ plate. Two weeks later, individually distinguishable colonies were visually identified and then transferred to a 96-well plate using a sterile pipette tip. Finally, single HEK293T cell clones exhibiting complete loss of endogenous PTER protein were confirmed by western blotting using a polyclonal anti-PTER antibody (Invitrogen, TR-1003-G).

### Western blotting

For analysis of samples from cell culture, cells were collected and lysed by probe sonication. Cell lysates were centrifuged at 13,000 r.p.m. for 10 min at 4 °C. The supernatant was collected and boiled for 10 min at 95 °C in 4× NuPAGE LDS sample buffer (Thermo Fisher, NP0008) supplemented with 100 mM DTT (Sigma, D0632-1G). For analysis of samples from mice, blood was obtained through submandibular bleeding using a 21 G needle (BD, 305129) into lithium heparin tubes (BD, 365985). Blood was subsequently spun down at 5,000 r.p.m. for 5 min at 4 °C to retrieve the supernatant plasma fractions. All tissue samples were dissected, weighed on a scale, collected into Eppendorf tubes and immediately frozen on dry ice and stored at −80 °C. A stereotaxic device was used to dissect out hypothalamus and brainstem. Adipose tissues were preserved in 4% paraformaldehyde (Fisher Scientific, AAJ19943K2) for histology analysis. Tissues were then mixed with 0.5 ml cold RIPA buffer and homogenized using a Benchmark BeadBlaster homogenizer at 4 °C. The mixture was spun down at 13,000 r.p.m. for 10 min at 4 °C to pellet the insoluble material. The supernatant was quantified using a tabletop Nanodrop One or using a BCA Protein Assay kit (Fisher Scientific, 23250) and analysed by western blotting. Adipose tissues from DIO mice were separately processed using a protein extraction kit to remove lipids (Invent Biotechnologies, AT-022). Proteins were separated on NuPAGE 4–12% Bis-Tris gels and transferred to nitrocellulose membranes. Equal loading was ensured by staining blots with Ponceau S solution. Blots were then incubated with Odyssey blocking buffer for 30 min at room temperature and incubated with primary antibodies (1:1,000 dilution rabbit anti-PTER antibody (Invitrogen, PA5-20750), 1:5,000 dilution rabbit anti-β-actin antibody (Abcam, ab8227), 1:1,000 dilution mouse anti-OxPhoS cocktail antibody (Invitrogen, 45-8099), 1:1,000 dilution rabbit anti-HSL antibody (Novus biologicals, NB110-37253), 1:1,000 dilution rabbit anti-pHSL (Novus biologicals, NBP3-05457), 1:1,000 dilution rabbit anti-ATGL (Cell Signaling, 2138), 1:5,000 dilution mouse anti-α-tubulin antibody (Cell Signaling, 3873S), 1:5,000 dilution mouse anti-Flag antibody (Sigma, F1804-200UG), 1:1,000 dilution rabbit anti-6×His antibody (Abcam, ab9108)) in blocking buffer overnight at 4 °C. Blots were washed three times with PBST (0.05% Tween-20 in PBS) and stained with species-matched secondary antibodies (1:10,000 dilution goat anti-rabbit IRDye 800RD (Li-Cor, 925-68070) and 1:10,000 dilution goat anti-mouse IRDye 680RD (Li-Cor, 925-68070)) at room temperature for 1 h. Blots were further washed three times with PBST and imaged with an Odyssey CLx Imaging System.

### Generation of recombinant mouse PTER proteins

The mouse *Pter* gene (UniProt Q60866) was codon optimized to ensure bacterial expression and was synthesized as gBlocks by IDT. The gene fragment was then inserted into a pET-20b vector containing a carboxy-terminal hexa-histidine (His) tag. DNA sequences encoding a Strep tag were cloned into the amino terminus of *Pter* for Strep-Tactin-based purification. BL21 competent bacteria (Thermo Scientific, EC0114) were used to transform pET-20b-mouse *Pter* plasmids and subsequently cultured in LB medium with ampicillin at 37 °C on a shaker overnight. BL21 cells were then transferred to autoinduction medium, which consisted of the following components: 10 g tryptone (Fisher Scientific, BP1421-500), 5 g yeast extract (Fisher Scientific, BP1422-500), 2 ml MgSO_4_ (1 M), 1 ml metal solution (0.05 M ferric citrate, 0.02 M CaCl_2_, 0.02 M ZnSO_4_, 2 µM CoCl_2_, 2 µM CuSO_4_, 2 µM NiCl_2_, 2 µM Na_2_MoO_4_ and 2 µM boric acid), 20 ml salt solution (167.5 g Na_2_HPO_4_, 85 g KH_2_PO_4_, 53.4 g NH_4_Cl and 17.8 g Na_2_SO_4_ in 500 ml water in total) and 20 ml sugar solution (125 g glycerol, 12.5 g glucose and 50 g α-lactose in 500 ml water in total) in a total volume of 1 litre. The bacteria were cultured until the optical density value reached a range of 0.5–0.7. Bacteria were subsequently incubated at 15 °C overnight before being spun down at 8,000 r.p.m. for 30 min at 4 °C. Bacteria were then lysed in PBS through probe sonication on ice to release cytosolic proteins. Soluble fractions were isolated by high-speed centrifugation at 15,000 r.p.m. for 30 min at 4 °C. They were then run down a nickel column using an ÄKTA pure chromatography system. The elution was performed from 0 mM to 300 mM NaCl in PBS over a gradient involving 60 column volumes. Fractions containing mouse PTER proteins were pooled together before undergoing another round of purification. This step involved running fractions down columns loaded with Strep-Tactin resins (IBA, 2-1208-002) following the manufacturer’s instructions. The bound PTER proteins were eluted by 2.5 mM d-desthiobiotin before passing through a HiPrep 16/60 Sephacryl S-200 size-exclusion column (Sigma, GE17-1166-01) in buffer containing 25 mM Tris and 100 mM NaCl. Finally, fractions containing monomeric PTER recombinant proteins were pooled together and subjected to SDS–PAGE gel electrophoresis to ensure >95% purity was achieved. The recombinant proteins were aliquoted and stored at −80 °C for subsequent enzymatic assays.

### Enzymatic assays

A total of 100 μg of proteins derived from cell or tissue lysates, or 100 ng of recombinant mouse PTER proteins or 50 µl of chromatography fractions were subjected to incubation in a 50 µl PBS solution (pH 7.4, 0.144 g l^–1^ KH_2_PO_4_, 9 g l^–1^ NaCl and 0.795 g l^–1^ Na_2_HPO_4_, Corning, 21-040-CV) at 37 °C for 1 h. For assays using kidney membrane and soluble fractions, total kidney homogenates were transferred into ultracentrifuge inserts and spun at 100,000*g* on a Beckman centrifuge I8-70M for 1 h at 4 °C. The supernatant was quantified as the kidney soluble fraction and the pellet was resuspended thoroughly in PBS and measured using a using a tabletop Nanodrop One. For assays using Tris-HCl buffer, the pH and salt composition was as follows: pH 7.4, 25 mM Tris-Cl. Next, 100 µM *N*-acetyltaurine (Cayman, 35169) was added for assaying hydrolysis. For assays testing the substrate scope of mouse PTER hydrolysis, 100 μM *N*-acetyl-l-isoleucine (Alfa Aesar, H66771), *N*-acetyl-l-methionine (Sigma, 01310-5G), *N*-acetyl-l-leucine (Sigma, 441511-25G), *N*-acetyl-l-valine (Alfa Aesar, H66943), *N*-acetyl-l-phenylalanine (Sigma, 857459-5G), *N*-acetyl-l-tyrosine (Sigma, PHR1173-1G), *N*-acetyl-l-serine (Sigma, A2638-1G), *N*-acetyl-l-proline (Sigma, A0783-1G), *N*-acetyl-l-threonine (Chem-Impex Int’l, 03262), *N*-acetyl-l-alanine (Sigma, A4625-1G), *N*-acetyl-β-alanine (Thermo Scientific, H50208.03), *N*-acetyl-l-arginine (Sigma, A3133-5G), *N*-acetyl-l-cysteine (Sigma, A7250-25G), *N*-acetyl-l-glutamic acid (Sigma, 855642-25G), *N*-acetyl-l-glutamine (Sigma, A9125-25G), *N*-acetyl-l-histidine (Alfa Aesar, J65657), *N*-acetyl-l-tryptophan (Sigma, A6376-10G), *N*-acetyl-glycine (Sigma, A16300-5G), *N*-acetyl-l-asparagine (Sigma, 441554-1G), *N*-acetyl-l-lysine (Sigma, A2010-1G), *N*-acetyl-l-aspartic acid (Sigma, 00920-5G), *N*-propionyl-taurine (Acme, AB38328), *N*-palmitoyl-taurine (Cayman, 10005611), *N*-oleoyl-taurine (Cayman, 10005609), *N*-stearoyl-taurine (Cayman, 10005610), *N*-arachidonoyl-taurine (Cayman, 10005537), taurolithocholic acid (Cayman, 17275), tauro-α-muricholic acid (Cayman, 20288) and taurocholic acid (Cayman, 16215) were used. For *N*-acetyltaurine synthesis, 10 mM taurine (Sigma, T0625-100G) and 10 mM acetate (Sigma, S2889-250G) were added. For assays testing the substrate scope of mouse PTER synthesis, 10 mM l-isoleucine (Sigma, I2752-1G), l-methionine (Sigma, M5308-25G), l-leucine (Sigma, L8000-25G), l-valine (Sigma, V-0500), l-phenylalanine (Alfa Aesar, A13238), l-tyrosine (Thermo Scientific, A11141.22), l-serine (Aldrich Chemical, S260-0), l-proline (Sigma, P0380-100G), l-threonine (Sigma, T8625-1G), l-alanine (Sigma, A7627-1G), β-alanine (Sigma, 05160-50G), l-arginine (Sigma, A5006-100G), l-cysteine (Sigma, 168149-25G), l-glutamic acid (Sigma, 49621-250G), l-glutamine (Sigma, G-3126), l-histidine (Sigma, H-8000), l-tryptophan (Sigma, T0254-5G), glycine (FisherChemical, G48-212), l-asparagine (Sigma, A0884-25G), l-lysine (Sigma, L5501-5G) and l-aspartic acid (United States Biochemical, 11625) were individually incubated with 10 mM acetate (Sigma, S2889-250G); 10 mM propionate (Sigma, P1880-100G), 10 mM butyrate (Sigma, B5887-1G) was incubated with 10 mM taurine; 1 mM palmitate (Sigma, P9767-5G), oleate (Sigma, O7501-1G), stearate (Sigma, S3381-5G), arachidonate (Sigma, 10931), lithocholate (Cayman, 20253), α-muricholate (Cayman, 20291) and taurocholate (Cayman, 16215) were individually incubated with 100 mM taurine. Reactions were then quenched and metabolites were extracted by 150 µl of a 2:1 mixture of acetonitrile and methanol. The mixture was spun down at 15,000 r.p.m. for 30 min at 4 °C. The supernatant was subsequently transferred to MS vials for LC–MS analysis.

### Molecular docking

The AlphaFold-predicted structure of mouse PTER (AF-Q60866-F1) was used to search for proteins with structural or sequence homology using FoldSeek and BLAST, respectively. The top-predicted structural match from the Protein Data Bank (PDB) database as identified by FoldSeek was PDB 3K2G, a resiniferatoxin-binding protein isolated from *Rhodobacter sphaeroides*. This crystal structure, along with annotation in UniProt, and metal binding-site prediction using MIB2, all indicated the presence of two zinc ions in the active site of PTER. Molecular docking was performed with CB-Dock2, an online docking server using curvature-based cavity prediction followed by AutoDock Vina-based molecular docking. The substrate compound *N*-acetyltaurine was prepared as a SDF file, and the AlphaFold-predicted protein structure for PTER was prepared as a PDB file. Ligand–receptor docking was performed using CB-Dock2 following the standard procedure. Ligand–receptor docking results were visually evaluated for biochemical feasibility, and docking results with the lowest Vina score were accepted. The predicted docking poses were evaluated using PyMol (v.3.7), and the predicted active-site residues were identified for mutation.

### Mouse PTER mutagenesis

A Q5 Site-Directed Mutagenesis kit (NEB, E0554S) was used to introduce mutations in amino acid residues predicted to have a role in stabilizing zinc ions, interacting with *N*-acetyltaurine or spatially constraining the active site of mouse PTER. The introduced mutations were subsequently verified through plasmid sequencing conducted by Genewiz.

### Activity-guided fractionation

A total of 6 kidneys from 10–14-week-old male C57BL/6J mice were homogenized using a Benchmark BeadBlaster homogenizer at 4 °C. The cytosolic fraction was obtained using high-speed centrifugation at 15,000 r.p.m. for 30 min at 4 °C. Then the mixture was concentrated using 3 kDa filter tubes (Millipore, UFC900324) by spinning down at 4,000 r.p.m. for 1 h. The concentrated sample was diluted 50× into buffer containing 20 mM Tris pH 7.5 before anion exchange on a 1 ml HiTrap Q column (Cytiva, GE29-0513-25). The elution was performed from 0 mM to 500 mM NaCl in 20 mM Tris pH 7.5 over a gradient involving 30 column volumes. Following anion exchange, each fraction was evaluated for *N*-acetyltaurine hydrolase activity as described above. Three fractions with the highest enzymatic activities were combined, concentrated and subjected to size exclusion on a Superose 6 Increase 10/300 GL column (Cytiva, GE29-0915-96). Each fraction from size exclusion was again evaluated for *N*-acetyltaurine hydrolase activity. The most active fraction was subjected to LC–MS analysis at the Vincent Coates Foundation Mass Spectrometry Laboratory, Stanford University Mass Spectrometry.

### Shotgun proteomics

Samples were reduced with 10 mM DTT for 20 min at 55 °C, cooled to room temperature and then alkylated with 30 mM acrylamide for 30 min. They were then acidified to a pH of about 1 with 2.6 µl of 27% phosphoric acid, dissolved in 165 µl of S-trap loading buffer (90% methanol and 10% 1 M triethylammonium bicarbonate (TEAB)) and loaded onto S-trap microcolumns (Protifi, C02-micro-80). After loading, the samples were washed sequentially with 150 µl increments of 90% methanol and 10% 100 mM TEAB, 90% methanol and 10% 20 mM TEAB, and 90% methanol and 10% 5 mM TEAB solutions, respectively. Samples were digested at 47 °C for 2 h with 600 ng of MS-grade Trypsin/LysC mix (Promega, V5113). The digested peptides were then eluted with two 35 µl increments of 0.2% formic acid in water and two more 40 µl increments of 80% acetonitrile with 0.2% formic acid in water. The four elutions were consolidated in 1.5 ml S-trap recovery tubes and dried by SpeedVac (Thermo Scientific). Finally, the dried peptides were reconstituted in 2% acetonitrile with 0.1% formic acid in water for LC–MS analysis.

MS experiments were performed using an Orbitrap Exploris 480 mass spectrometer (Thermo Scientific) attached to an Acquity M-Class UPLC system (Waters). The UPLC system was set to a flow rate of 300 nl min^–1^, for which mobile phase A was 0.2% formic acid in water and mobile phase B was 0.2% formic acid in acetonitrile. The analytical column was prepared in-house with an inner diameter of 100 µm pulled to a nanospray emitter using a P2000 laser puller (Sutter Instrument). The column was packed with Dr. Maisch 1.9 µm C18 stationary phase to a length of approximately 25 cm. Peptides were directly injected onto the column with a gradient of 3–45% mobile phase B, followed by a high-B wash over a total of 80 min. The mass spectrometer was operated in a data-dependent mode using HCD fragmentation for MS/MS spectra generation.

RAW data were analysed using Byonic (v.4.4.1; Protein Metrics) to identify peptides and to infer proteins. A concatenated FASTA file containing UniProt *Mus musculus* proteins and other probable contaminants and impurities was used to generate an in silico peptide library. Proteolysis with Trypsin/LysC was assumed to be semi-specific allowing for N-ragged cleavage with up to two missed cleavage sites. Both precursor and fragment mass accuracies were held within 12 ppm. Cysteine modified with propionamide was set as a fixed modification in the search. Variable modifications included oxidation on methionine, histidine and tryptophan, dioxidation on methionine and tryptophan, deamidation on glutamine and asparagine, and acetylation on protein N terminus. Proteins were held to a false discovery rate of 1% using standard reverse-decoy technique. Overall, 247 proteins with at least 1 peptide matched in total (Supplementary Table 1). PTER ranked number 6 on the list.

### Preparation of mouse tissues for LC–MS analysis

Plasma (50 µl) was mixed with 150 µl of a 2:1 mixture of acetonitrile and methanol and vortexed for 30 s. The mixture was centrifuged at 15,000 r.p.m. for 10 min at 4 °C and the supernatant was transferred to a LC–MS vial. For other mouse tissues, 50 µg of sample was mixed with 150 µl of a 2:1 mixture of acetonitrile and methanol and homogenized using a Benchmark BeadBlaster homogenizer at 4 °C. The mixture was spun down at 13,000 r.p.m. for 10 min at 4 °C to pellet the insoluble material. The supernatant was then transferred to a LC–MS vial.

### Measurements of metabolites by LC–MS

Metabolite measurements were performed using an Agilent 6520 Quadrupole time-of-flight LC–MS instrument as previously described^29^. MS analysis was performed using electrospray ionization (ESI) in negative mode. The dual ESI source parameters were configured as follows: the gas temperature was maintained at 250 °C with a drying gas flow of 12 l min^–1^ and the nebulizer pressure at 20 p.s.i.; the capillary voltage was set to 3,500 V; and the fragmentor voltage set to 100 V. The separation of polar metabolites was conducted using a Luna 5 μm NH2 100 Å LC column (Phenomenex 00B-4378-E0) with normal phase chromatography. Mobile phases were as follows: buffer A, 95:5 water and acetonitrile with 0.2% ammonium hydroxide and 10 mM ammonium acetate; buffer B, acetonitrile. The LC gradient was initiated at 100% B with a flow rate of 0.2 ml min^–1^ from 0 to 2 min. The gradient was then linearly increased to 50% A/50% B at a flow rate of 0.7 ml min^–1^ from 2 to 20 min. From 20 to 25 min, the gradient was maintained at 50% A/50% B at a flow rate of 0.7 ml min^–1^. *N*-acetyltaurine (Cayman, 35169) eluted around 12 min and taurine (Sigma, T0625-500G) was eluted around 13 min under the above conditions. The list of metabolites detected using LC–MS is summarized in Supplementary Table 2. Metabolite data were analysed using Agilent Qualitative Analysis software (v.B.07.00).

### General animal information

All animal experiments were performed according to protocols approved by the Stanford University Administrative Panel on Laboratory Animal Care. Mice were maintained in 12-h light–dark cycles at 22 °C and about 50% relative humidity and fed a standard irradiated rodent chow diet. Where indicated, a high-fat diet (D12492, Research Diets 60% kcal from fat) was used. Male C57BL/6J (stock number 000664), male C57BL/6J DIO mice (stock number 380050) and male *Mc4r* KO mice (stock number 032518) were purchased from the Jackson Laboratory. Whole-body *Pter* KO mice (catalogue number C57BL/6N(Jax)-Pter^em1(IMPC)Bay^) were obtained from the Baylor KOMP2 group of International Mouse Phenotyping Consortium. For intraperitoneal injections of mice with compounds, compounds were dissolved in saline (Teknova, S5825). Compounds were administered to mice every day by intraperitoneal injections at 10 μl g^–1^ body weight at the indicated doses. For chronic intraperitoneal injection, oral gavage and subcutaneous injection experiments, mice were mock treated with saline for 3–5 days until body weights were stabilized. For control IgG or anti-GFRAL antibody treatment, mice were subcutaneously injected with 10 mg kg^–1^ antibodies once every 3 days. For GLP-1 and exendin-3 injection, GLP-1 and exendin-3 powder was first dissolved in 18:1:1 saline, DMSO and kolliphore and then injected (GLP-1: 2 mg per kg per day, i.p.; exendin-3, 0.1 mg per kg per day, i.p.). Unless specified, compounds were administered at a time of around 18:00. For measuring known feeding-regulating polypeptide hormones, blood plasma was collected at 9:00 and ELISA kits were used following manufacturer’s instructions (leptin: Crystal Chem, 90030; GLP-1: Sigma, EZGLP1T-36K; GDF15: R&D Systems, MGD150; adiponectin: Crystal Chem, 80569; FABP4: Novus Biologicals, NBP2-82410; insulin: Crystal Chem, 90080; ALT: Cayman, 700260; AST: Cayman, 701640; triglycerides: Cayman, 10010303; and NEFA: Cayman, 700310).

### Breeding and genotyping of *Pter* KO mice

*Pter* KO and WT animals were generated through heterozygous breeding crosses and weaned around postnatal day 21. Genotyping was performed using the following procedures: tail clippings were collected from littermates and boiled for 30 min at 95 °C in 100 μl of 50 mM NaOH to extract genomic DNA. The solution was neutralized by adding 42 μl of 0.5 M Tris (pH 7.5). PCRs were performed by using primers for either the *Pter* WT allele (forward, 5′-TCATGTCCCACCTTGACAGGTAAGCGGGTC-3′; reverse, 5′-CAGTTGTAGCAGCCATGAACA CTATTGTGC-3′) or *Pter* KO allele (forward, 5′-GGGTAATATACTTGTCAAACCATGCT-3′; reverse, 5′-CAGTTGTAGCAGCCATGAACA-3′). Promega GoTaq master mix (Promega, PRM7123) was used for the PCR reaction. Each 25 μl reaction consisted of 12.5 μl of the Promega master mix, 2.5 μl of a 10 μM mixture of forward and reverse primers, 2 μl of genomic DNA and 8 μl of ultrapure water. The thermocycling program on a Bio-Rad C1000 Touch Thermo Cycler began with an initial 90 s at 98 °C, followed by cycles of 30 s at 98 °C, 30 s at 58 °C for KO primers and 50 °C for WT primers and 30 s at 72 °C, followed by 5 min at 72 °C and finally held at 4 °C. PCRs for WT primers consisted of 41 cycles, whereas PCRs for KO primers consisted of 35 cycles. Samples were run on a 1.5% agarose gel with 0.1 mg ml^–1^ ethidium bromide. WT alleles were expected to produce a PCR product of 699 bp in size, whereas KO alleles were expected to produce PCR products that are 479 bp in size.

### Taurine water supplementation

Taurine (2.5% (w/v); Sigma, T0625-500G) was dissolved in mouse drinking water and given to 4-week-old male *Pter* KO mice and WT mice. Taurine water was freshly prepared every 3 days while mice were on a high-fat diet (D12492, Research Diets 60% kcal from fat). Body weights, food intake and water consumption were measured every 3 days. No adverse effects were observed in mice fed with taurine water.

### *N*-acetyltaurine ex vivo kinetic analysis

Kidneys from 8-week-old *Pter* KO mice and WT mice were dissected and incubated with 9× (v/w) pre-warmed Williams Medium E (Quality Biological, 112-033-101) supplemented with 5 µM heavy *N*-acetyltaurine (Acme) at 37 °C on a shaker. Supernatant medium (30 µl) was collected at 0, 15, 30, 45, 60, 90, 120 and 240 min of incubation. Metabolites were extracted and analysed by LC–MS as described above.

### Adipose lipolysis in vivo and ex vivo

Blood plasma and epidydimal fat were collected from 4-month-old male DIO C57BL/6J mice receiving saline, *N*-acetyltaurine (15 mg kg^–1^, i.p.) or noradrenaline (0.5 mg kg^–1^, i.p.) treatment. Blood glycerol contents were determined using a glycerol quantification kit (Sigma, F6428-40ML). For mature adipocyte lipolysis ex vivo, epidydimal fat from 4-month-old male DIO C57BL/6J mice was dissected and dissociated using 2 mg ml^–1^ collagenase B (Worthington, CLSAFB) and 1 mg ml^–1^ soybean trypsin inhibitor (Worthington, LS003570). Digested adipose tissues were spun down at 500*g* for 3 min to isolate the floating layer of mature adipocytes. Around 1 million mature adipocytes were collected and incubated with saline, 50 μM *N*-acetyltaurine or 1 μM noradrenaline at 37 °C on a shaker for 1 h. Then released glycerol was determined using a glycerol quantification kit (Sigma, F6428-40ML).

### Indirect calorimetry and physiological measurements

Male *Pter* KO mice and WT mice (8–9 weeks old; *N* = 9 per group) were supplemented with 2.5% (w/v) taurine water and fed on a high-fat diet for 4 weeks. Taurine water was freshly prepared every 3 days when body weights and food intake were measured. Before the body weights of *Pter* KO mice started to be significantly different from WT mice (4 weeks on taurine water), metabolic parameters including oxygen consumption, carbon dioxide production, RER, food intake and ambulatory movement of mice were measured using the environment-controlled home-cage CLAMS system (Columbus Instruments) at the Stanford Diabetes Center. A separate group of 12–13-week-old male *Pter* KO mice and WT mice (*N* = 8 per group) were supplemented with 2.5% (w/v) taurine water and fed on a high-fat diet for 8 weeks before placement into the metabolic cages for analysis. Mice were housed in the metabolic chambers for 36 h before the start of the experiment. Data collected during a complete 24-h day–night cycle were used for analysis. Energy expenditure calculations were normalized for body weight. *P* values were calculated from two-tailed unpaired *t*-tests.

### Mouse exercise training protocols

A Columbus Instrument animal treadmill with six lanes (Columbus, 1055-SRM-D65) was used for the treadmill running experiments. Before commencing the treadmill running, mice were given a 5-min acclimation period. The initial treadmill running phase began at a speed of 7.5 m min^–1^ with a 4° incline, following a previously described procedure^29^. At intervals of 3 min, both the speed and incline were incrementally increased by 2.5 m min^–1^ and 2°, respectively. Once the maximum parameters of 40 m min^–1^ in speed and a 30° incline were attained, they remained constant until the mice reached a state of exhaustion, defined as when the mice remained on the shocker at the rear of the treadmill for longer than 5 s. *Pter* KO mice and WT mice were exercised every other day while on a high-fat diet (60% kcal from fat) for a duration of 6 weeks. Running was performed in the mid-morning for all experiments. Body weights and food intake were measured immediately before each exercise training session.

### Glucose tolerance and insulin tolerance tests in mice

For glucose tolerance tests, mice were fasted for 6 h (fasting starting at 7:00 in the morning) and then i.p. injected with glucose at 2 g kg^–1^ body weight. Blood glucose levels were measured at 0, 20, 40, 60 and 120 min by tail bleeding using a glucose meter. For insulin tolerance tests, mice were fasted for 6 h (fasting starting at 7:00 in the morning) and then i.p. injected with insulin in saline 0.75 U kg^–1^ body weight. Blood glucose levels were measured at 0, 20, 40, 60 and 120 min by tail bleeding using a glucose meter.

### hCom2 bacterial strains and culture conditions

Individually cultivated hCom2 strains were obtained from the Microbiome Therapies Initiative. All strains were cultured in one of two growth medium: mega medium and chopped meat medium with rumen fluid and carbohydrates. Cultures were incubated at 37 °C in an anaerobic chamber (Coy Laboratories) in an atmosphere of 5% hydrogen, 10% CO_2_ and 85% N_2_. Cultures were stored in anaerobically prepared 25% glycerol and water (v/v). All medium and reagents used in the anaerobic chamber were pre-reduced for at least 48 h.

### Synthetic community construction

Frozen stocks in 96-well plate matrix tubes were thawed, and 300 µl of each thawed culture was used to inoculate 40 ml of growth medium in 50 ml Falcon tubes. After 72 h, non-normalized cultures of all strains were pooled into a mixture. A 1 ml aliquot of the resulting mixed culture was stored at −80 °C for metagenomic sequencing. The remainder of the mixed culture was subjected to centrifugation (4,700*g*, 30 min). The cell pellet was washed with an equal volume of pre-reduced sterile PBS and then resuspended in 1/120 of the initial volume of 25% glycerol and water (v/v) solution. Aliquots of the resulting synthetic community were stored in 2 ml cryovials (Corning, 430659) at −80 °C until use.

### Gnotobiotic mouse experiments

Germ-free C57BL/6N mice (male, 6–8 weeks of age) were originally obtained from Taconic Biosciences and colonies were maintained in gnotobiotic isolators and fed ad libitum. The Institutional Animal Care and Use Committee at Stanford University approved all procedures involving animals. Glycerol stocks of synthetic communities were thawed and shaken well at room temperature, and mice were orally gavaged with 200 µl of the mixed culture. To ensure efficient colonization by all strains in the community, mice were gavaged using the same procedure twice on different days for all experiments. Mice were fed standard chow (LabDiet, 5k67), fresh faecal pellets were collected weekly at the same time of day and stored at −80 °C before analysis. The mice were maintained on a standard diet (LabDiet, 5k67; 0.2% Trp) for 4 weeks before euthanasia (fed ad libitum). Fresh faecal samples from germ-free mice and hCom2-colonized mice were collected, normalized by weight, homogenized and spun down to isolate live bacteria for in vitro incubation. Mice were euthanized humanely by CO_2_ asphyxiation and the plasma was collected in a BD blood tube (BD 365967) and stored on ice. Plasma samples were centrifuged at 16,000*g* for 20 min, and supernatant was stored in −80 °C until use.

### hCom2 in vitro screening

Individually cultivated hCom2 strains were resuspended in a standard amino acid complete (SAAC) medium as previously described^36^. A volume of 100 µl of cell suspension from each strain was incubated with 10 mM taurine and 10 mM acetate in 300 µl SAAC medium in an anaerobic chamber (Coy Laboratories) in an atmosphere of 5% hydrogen, 10% CO_2_ and 85% N_2_. Cells were spun down after 48 h of incubation to obtain cell pellets and conditioned medium. Metabolites were extracted and analysed by LC–MS. The optical density at 600 nm before and after incubation was measured. The list of strains screened can be found in Supplementary Table 3.

### Antibiotic treatment in mice

Mice (12–14 weeks old) were treated with antibiotic mixture (chloramphenicol (Sigma, C0378-25G), spectinomycin dihydrochloride pentahydrate (Sigma, S4014-25G), apramycin sulfate (Sigma, A2024-5G), tetracycline hydrochloride (Sigma, T7660-5G), kanamycin (Thermo Scientific, 11815032) and ampicillin (Sigma, A9518) at 1 g l^–1^ per antibiotic) was administered in drinking water ad libitum and orally gavaged (0.5 ml) every other day for a duration of 2 weeks. Before blood was collected from these mice, fresh faecal samples were collected using sterile pre-weighted Eppendorf tubes and labelled with unique identifiers. Samples were immediately stored at −80 °C until further processing. Faecal samples were normalized by weight, homogenized and filtered before DNA extraction. DNA was extracted from faecal samples using a Qiagen Mini Prep kit following the manufacturer’s protocol. Extracted DNA was stored at −20 °C until quantitative PCR analysis.

Universal bacterial primers targeting the V3 region of the bacterial 16S rRNA were selected (forward HV3-16S primer: 5′CCAGACTCCTACGGGAGGCAG-3′; reverse HV3-16S primer: 5′-CGTATTACCGCGGCTGCTG-3′). Mouse genomic DNA was used as housekeeping gene for quantitative PCR analysis. All reactions were carried out with 10 ng total DNA and SsoAdvanced Universal SYBR Green Supermix (Bio-Rad, 1725274) in CFX Opus 384. Reactions were held at 95 °C for 10 min, followed by 40 cycles of 95 °C for 15 s and 60 °C for 60 s. The number of 16s DNA copies was subsequently determined and normalized to the number of mouse genomic DNA copies in the same faecal sample.

### Reporting summary

Further information on research design is available in the Nature Portfolio Reporting Summary linked to this article.

## Online content

Any methods, additional references, Nature Portfolio reporting summaries, source data, extended data, supplementary information, acknowledgements, peer review information; details of author contributions and competing interests; and statements of data and code availability are available at 10.1038/s41586-024-07801-6.

## Supplementary information

Supplementary Fig. 1 Unprocessed original images of gels and western blots. Uncropped western blots corresponding to the indicated figure panels, with molecular weight marker, lanes and primary antibody indicated.

Reporting Summary

Supplementary Table 1 Shotgun proteomics data of active HPLC fractions containing *N*-acetyltaurine deacetylase activity. Table of proteins identified in the active kidney cytosolic fraction, ranked by Byonic *P* value.

Supplementary Table 2 Mass-to-charge ratios and retention times of metabolites detected in this study. Table of MS parameters used for identification of the indicated metabolites.

Supplementary Table 3 List of hCom2 bacterial strains screened in vitro for *N*-acetyltaurine synthesis activity. Table of individual bacterial strains from hCom2 tested for in vitro synthesis activity.

## Source data

Source Data Fig. 1

Source Data Fig. 2

Source Data Fig. 3

Source Data Fig. 4

Source Data Fig. 5

Source Data Extended Data Fig. 1

Source Data Extended Data Fig. 2

Source Data Extended Data Fig. 3

Source Data Extended Data Fig. 4

Source Data Extended Data Fig. 5

Source Data Extended Data Fig. 6

Source Data Extended Data Fig. 7

Source Data Extended Data Fig. 8

Source Data Extended Data Fig. 9

Source Data Extended Data Fig. 10

## Data Availability

All data generated or analysed during this study are included in this article and its supplementary information files. Associated metabolomics data have been deposited into the EMBL-EBI MetaboLights database with the identifier MTBLS10408. Source data are provided with this paper.

## Code availability

No new code was generated in this study.
