# Supplementary material for: PTER is a N-acetyltaurine hydrolase that regulates feeding and obesity
Source: Nature. 2024 Aug 7;633(8028):182–8. doi: 10.1038/s41586-024-07801-6 (PMC11374699; doi:10.1038/s41586-024-07801-6)

nature portfolio

https://doi.org/10.1038/s41586-024-07801-6

Supplementary information

PTER is a *N*-acetyltaurine hydrolase that regulates feeding and obesity

In the format provided by the
authors and unedited

Fig. 1g

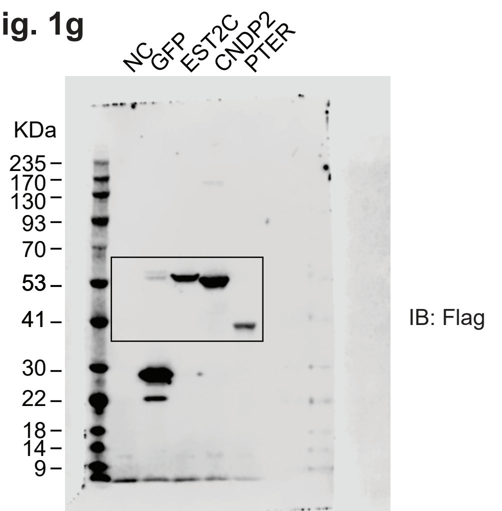

Fig. 1h

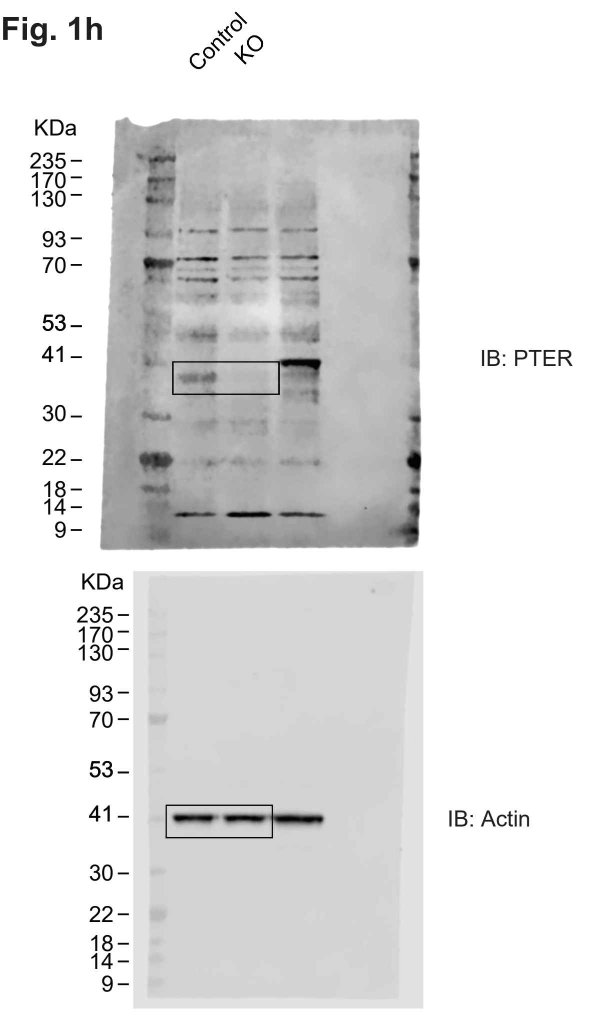

Fig. 2d

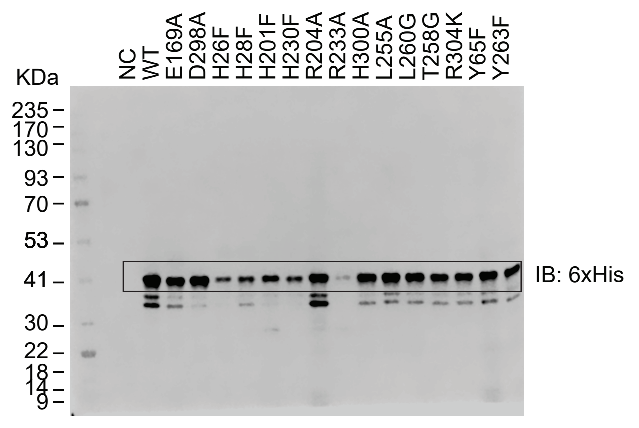

Fig. 3a

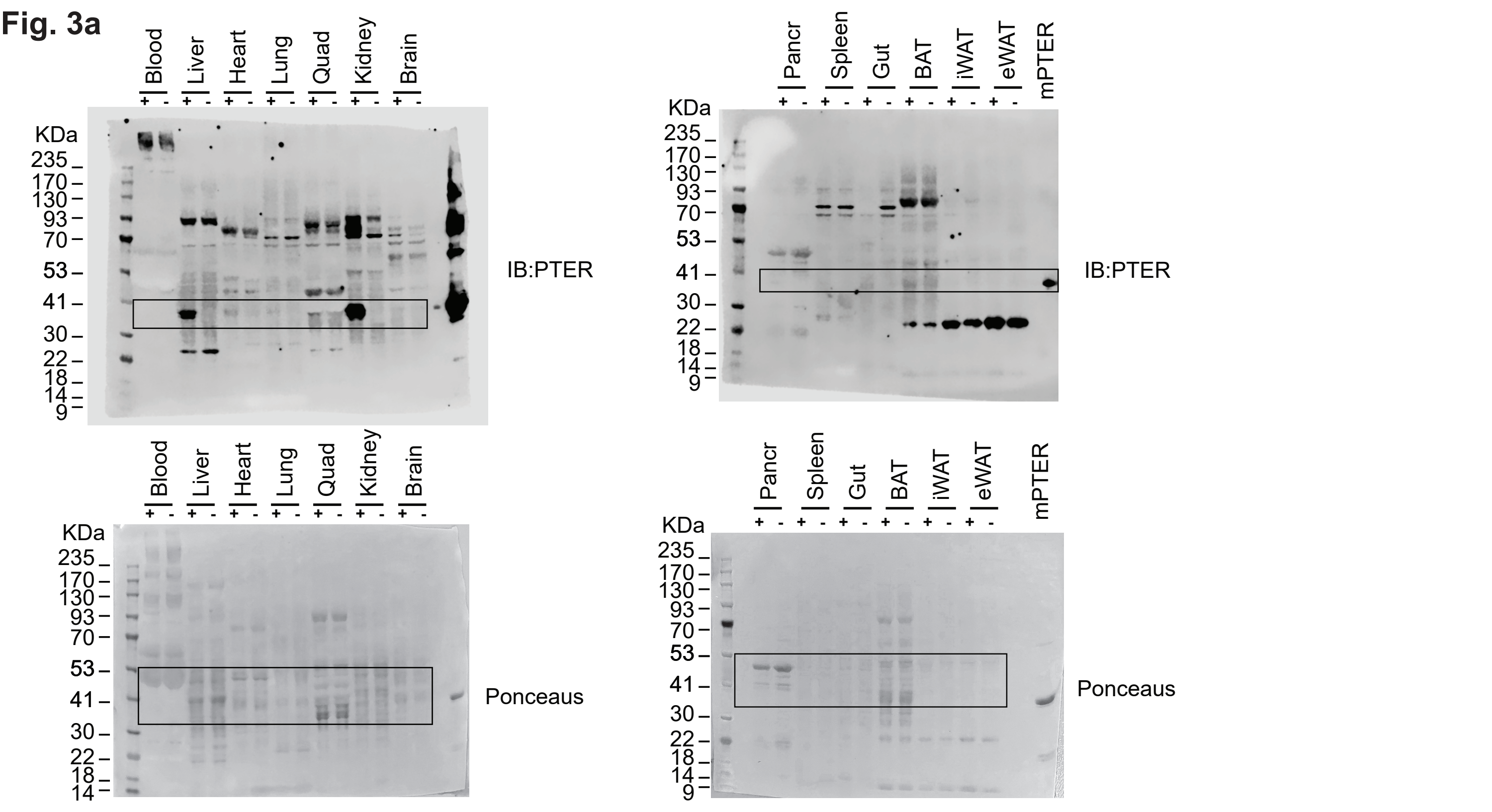

Fig. 5e

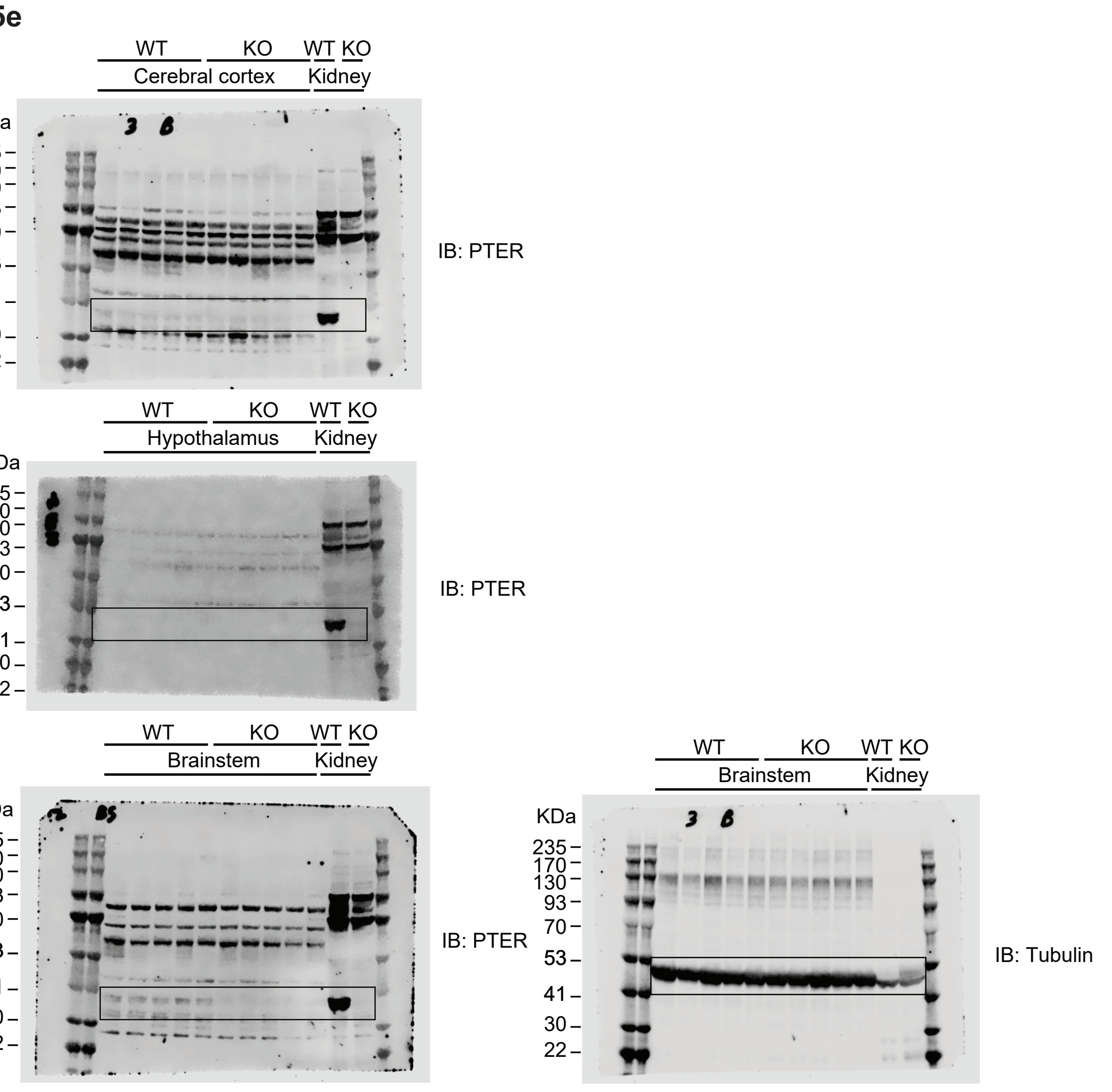

Extended Data Fig. S4c

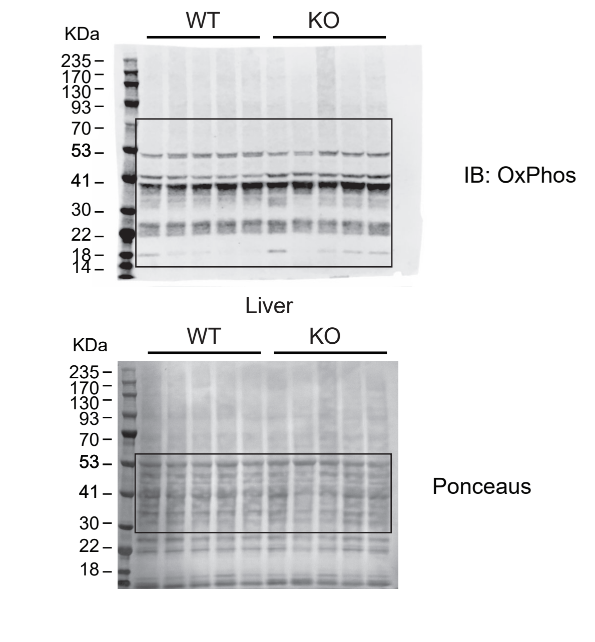

Extended Data Fig. S4d

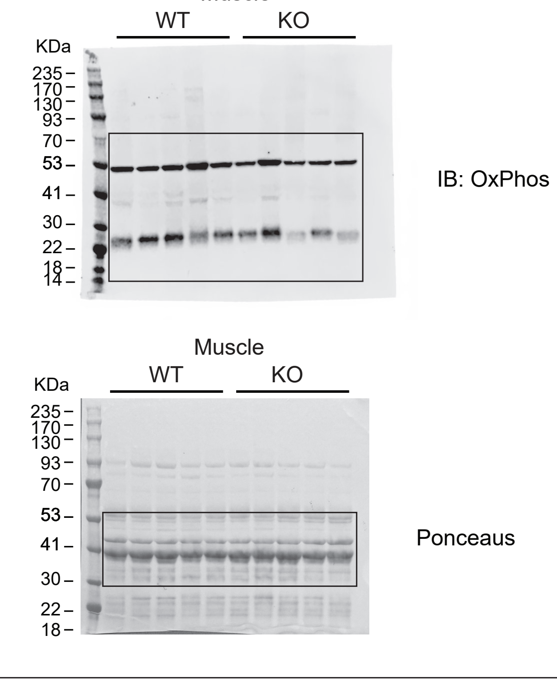

Extended Data Fig. S9l

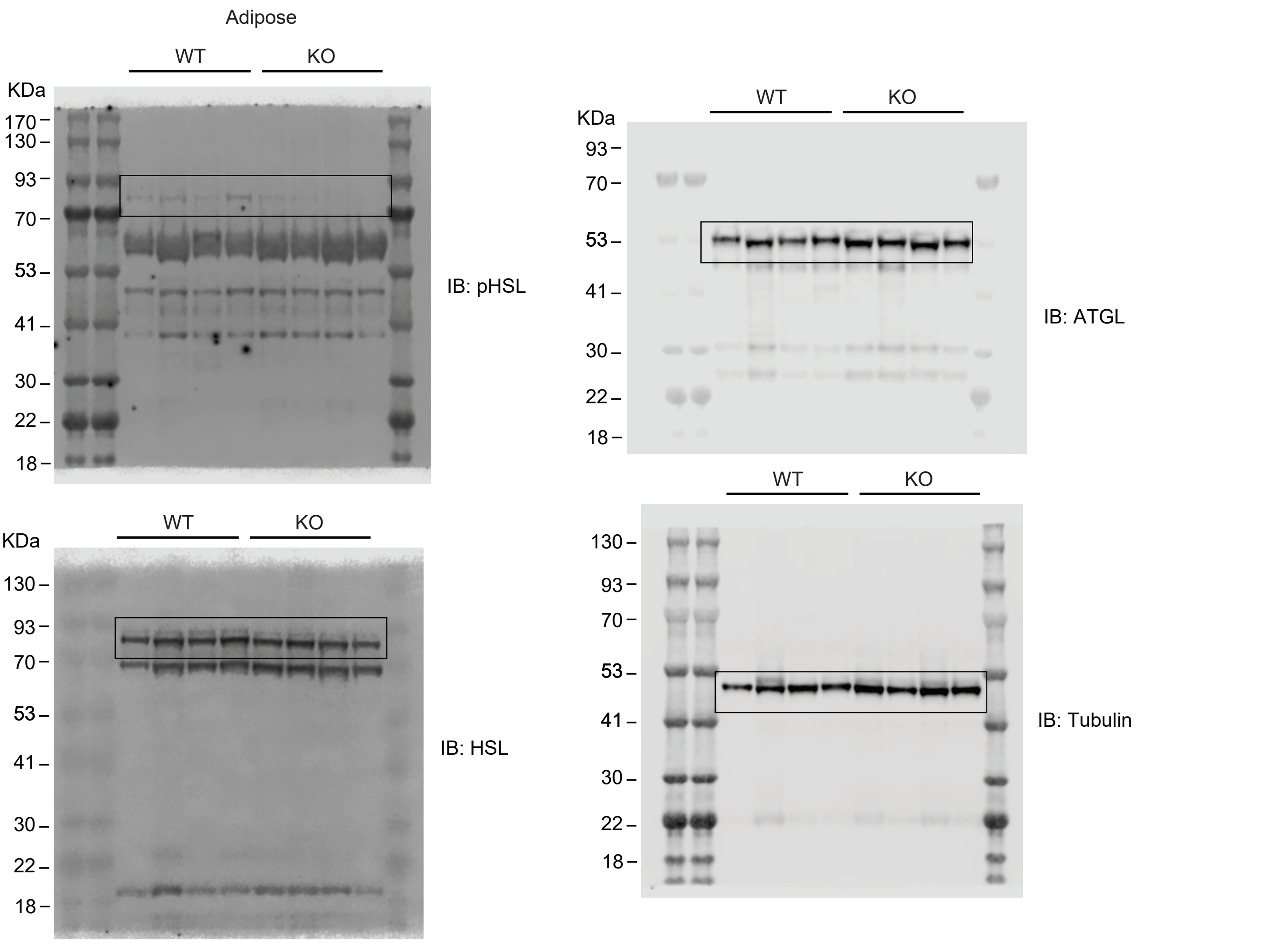

Supplement: Supplementary file 1 — Unprocessed original images of gels and western blots. Uncropped western blots corresponding to the indicated figure panels, with molecular weight marker, lanes and primary antibody indicated. [file 41586_2024_7801_MOESM1_ESM.pdf]
