# Supplementary material for: PTER is a N-acetyltaurine hydrolase that regulates feeding and obesity
Source: Nature. 2024 Aug 7;633(8028):182–8. doi: 10.1038/s41586-024-07801-6 (PMC11374699; doi:10.1038/s41586-024-07801-6)
Supplement: Supplementary file 2 — Reporting Summary [file 41586_2024_7801_MOESM2_ESM.pdf]

nature portfolio

Corresponding author(s): Jonathan Z. Long

Last updated by author(s): Jun 11, 2024

Reporting Summary

Nature Portfolio wishes to improve the reproducibility of the work that we publish. This form provides structure for consistency and transparency in reporting. For further information on Nature Portfolio policies, see our Editorial Policies and the Editorial Policy Checklist.

Statistics

For all statistical analyses, confirm that the following items are present in the figure legend, table legend, main text, or Methods section.

n/a	Confirmed	
☐	☒	The exact sample size (*n*) for each experimental group/condition, given as a discrete number and unit of measurement
☐	☒	A statement on whether measurements were taken from distinct samples or whether the same sample was measured repeatedly
☐	☒	The statistical test(s) used AND whether they are one- or two-sided<br>*Only common tests should be described solely by name; describe more complex techniques in the Methods section.*
☒	☐	A description of all covariates tested
☒	☐	A description of any assumptions or corrections, such as tests of normality and adjustment for multiple comparisons
☐	☒	A full description of the statistical parameters including central tendency (e.g. means) or other basic estimates (e.g. regression coefficient) AND variation (e.g. standard deviation) or associated estimates of uncertainty (e.g. confidence intervals)
☐	☒	For null hypothesis testing, the test statistic (e.g. *F*, *t*, *r*) with confidence intervals, effect sizes, degrees of freedom and *P* value noted<br>*Give P values as exact values whenever suitable.*
☒	☐	For Bayesian analysis, information on the choice of priors and Markov chain Monte Carlo settings
☒	☐	For hierarchical and complex designs, identification of the appropriate level for tests and full reporting of outcomes
☒	☐	Estimates of effect sizes (e.g. Cohen's *d*, Pearson's *r*), indicating how they were calculated

Our web collection on statistics for biologists contains articles on many of the points above.

Software and code

Policy information about availability of computer code

Data collection	Measurements of metabolites were conducted on an Agilent QTOF 6520 or QQQ mass spectrometer using Agilent's LC/MS Data Acquisition software (version B.08.00). Shotgun proteomics were performed on an Orbitrap Exploris 480 mass spectrometer.
Data analysis	Metabolite data were analyzed using Agilent Qualitative Analysis software (version B.07.00). Proteomic data were analyzed using Byonic (version 4.4.1). Data were displayed using Prism version 8.0 and R Studio (version 2021.9.1.372).

For manuscripts utilizing custom algorithms or software that are central to the research but not yet described in published literature, software must be made available to editors and reviewers. We strongly encourage code deposition in a community repository (e.g. GitHub). See the Nature Portfolio guidelines for submitting code & software for further information.

Data

Policy information about availability of data

All manuscripts must include a data availability statement. This statement should provide the following information, where applicable:

- Accession codes, unique identifiers, or web links for publicly available datasets
- A description of any restrictions on data availability
- For clinical datasets or third party data, please ensure that the statement adheres to our policy

All data generated or analyzed during this study are included in this published article and its supplementary information files. Source data are provided with this paper. Metabolomics data associated have been deposited to MetaboLights database (study number MTBLS10408).

Research involving human participants, their data, or biological material

Policy information about studies with human participants or human data. See also policy information about sex, gender (identity/presentation), and sexual orientation and race, ethnicity and racism.

Reporting on sex and gender	Not applicable
Reporting on race, ethnicity, or other socially relevant groupings	Not applicable
Population characteristics	Not applicable
Recruitment	Not applicable
Ethics oversight	Not applicable

Note that full information on the approval of the study protocol must also be provided in the manuscript.

Field-specific reporting

Please select the one below that is the best fit for your research. If you are not sure, read the appropriate sections before making your selection.

☒ Life sciences ☐ Behavioural & social sciences ☐ Ecological, evolutionary & environmental sciences

For a reference copy of the document with all sections, see nature.com/documents/nr-reporting-summary-flat.pdf

Life sciences study design

All studies must disclose on these points even when the disclosure is negative.

Sample size	Statistical tests were not employed to predetermine the sample size. The selection of sample sizes was guided by prior literature in the field of metabolism that had conducted similar experiments and by the number of replicates sufficient to detect outlier samples. For in vitro experiments, sample sizes ranged from 3 to 5 biological replicates, while for animal experiments, sample sizes of greater than 5 were used. The exact numbers were pre-determined based on the experimental design, the availability of animals, and the animal housing conditions, as well as previous relevant literature precedent (PMID: 33981039; 36921622).
Data exclusions	Animals were excluded from in vivo experiments based on body weights or body weight changes that exceeded three standard deviations.
Replication	The number of replicates is specified in each of the figure legends.
Randomization	For animal experiments involving the same genotype of mice (compound injection experiments), mice were randomly assigned to the study group. For experiments involving PTER-KO and WT animals, mice were assigned to each group based on genotype. Mice were age and sex-matched in all experiments. For molecular and cellular experiments (in vitro enzymatic assays using cell lysates and recombinant proteins), samples were randomly assigned to the study group.
Blinding	In all experiments, blinding of investigators was not implemented due to the quantitative nature of the measurements. All samples and experiments were performed in the way regardless of group or treatment.

Reporting for specific materials, systems and methods

We require information from authors about some types of materials, experimental systems and methods used in many studies. Here, indicate whether each material, system or method listed is relevant to your study. If you are not sure if a list item applies to your research, read the appropriate section before selecting a response.

Materials & experimental systems

n/a	Involved in the study	
☐	☒	Antibodies
☐	☒	Eukaryotic cell lines
☒	☐	Palaeontology and archaeology
☐	☒	Animals and other organisms
☒	☐	Clinical data
☒	☐	Dual use research of concern
☒	☐	Plants

Methods

n/a	Involved in the study	
☒	☐	ChIP-seq
☒	☐	Flow cytometry
☒	☐	MRI-based neuroimaging

Antibodies

Antibodies used	1:1000 dilution rabbit anti-PTER antibody (Invitrogen, PA5-20750), 1:5000 dilution rabbit anti-β-actin antibody (Abcam, ab8227), 1:5000 dilution mouse anti-Flag antibody (Sigma, F1804-200UG), 1:1000 dilution rabbit anti-6xHis antibody (Abcam, ab9108), 1:10000 dilution goat anti-rabbit IRDye 800RD (LI-COR, 925-68070) and 1:10000 dilution goat anti-mouse IRDye 680RD (LI-COR, 925-68070), 1:1000 dilution mouse anti-OxPhoS cocktail antibody (Invitrogen, 45-8099), 1:1000 dilution rabbit anti-HSL antibody (Novus biologicals, NB110-37253), 1:1000 dilution rabbit anti-pHSL (Novus biologicals, NBP3-05457), 1:1000 dilution rabbit anti-ATGL (Cell signaling, 2138), 1:5000 dilution mouse anti-alpha-tubulin antibody (Cell Signaling, 3873S), 10 mg/kg, IP, anti-GFRAL antibody (clone 8A2) was provided by Paul Emmerson, Eli Lilly & Co.
Validation	All antibodies used in this study are commercially available. Information on antibody validation can be found on the manufacturer's website. Links for each antibody are shown below.<br>1. Rabbit anti-PTER antibody (https://www.thermofisher.com/antibody/product/PTER-Antibody-Polyclonal/PA5-20750)<br>2. Rabbit anti-β-actin antibody (https://www.abcam.com/products/primary-antibodies/beta-actin-antibody-ab8227.html)<br>3. Mouse anti-Flag antibody (https://www.sigmaaldrich.com/US/en/product/sigma/f1804)<br>4. Rabbit anti-6xHis antibody (https://www.abcam.com/products/primary-antibodies/6x-his-tag-antibody-ab9108.html)<br>5. Goat anti-rabbit IRDye 800RD (https://www.licor.com/bio/reagents/irdye-680rd-goat-anti-mouse-igg-secondary-antibody)<br>6. Goat anti-mouse IRDye 680RD (https://www.licor.com/bio/reagents/irdye-680rd-goat-anti-mouse-igg-secondary-antibody)<br>7. Mouse anti-OXPHOS antibody (https://www.thermofisher.com/antibody/product/OxPhos-Rodent-WB-Antibody-clone-Cocktail-Cocktail/45-8099)<br>8. Rabbit anti-HSL antibody (https://www.novusbio.com/products/hormone-sensitive-lipase-hsl-antibody_nb110-37253)<br>9. Rabbit anti-pHSL antibody (https://www.novusbio.com/products/hormone-sensitive-lipase-hsl-antibody_nbp3-05457)<br>10. Rabbit anti-ATGL antibody (https://www.cellsignal.com/products/primary-antibodies/atgl-antibody/2138)<br>11. Mouse anti-alpha-tubulin antibody (https://www.cellsignal.com/products/primary-antibodies/a-tubulin-dm1a-mouse-mab/3873)<br>For the anti-GFRAL antibody, we validated by loss of the anorexigenic effect of recombinant GDF15 in mice.

Eukaryotic cell lines

Policy information about cell lines and Sex and Gender in Research

Cell line source(s)	HEK293T cell line (CRL-3216) from American Type Culture Collection (ATCC)
Authentication	The HEK293T cell line used in this study was authenticated by ATCC.
Mycoplasma contamination	The cell line was negative for mycoplasma contamination.
Commonly misidentified lines (See ICLAC register)	No commonly misidentified cell lines were used in this study

Animals and other research organisms

Policy information about studies involving animals; ARRIVE guidelines recommended for reporting animal research, and Sex and Gender in Research

Laboratory animals	Experiments were performed in the following mouse strains:<br>1. WT male C57BL/6J mice from Jackson Laboratory (stock no. 000664) at 10 to 14 weeks old.<br>2. WT male diet-induced obese C57BL/6J mice from Jackson Laboratory (stock no. 380050) from 17 to 28 weeks old.<br>3. Male whole-body PTER-KO mice (catalogue number, C57BL/6N(Jax)-Pterem1(IMPC)Bay) from the Baylor KOMP2 group of International Mouse Phenotyping Consortium (IMPC)) at 4 to 14 weeks old.<br>4. Male MC4R KO mice from Jackson Laboratory (stock no. 032518) at 12-16 weeks old.
Wild animals	Wild animals were not used in this study.
Reporting on sex	Only male mice were used in this study. Sex was not considered in study design.
Field-collected samples	Field-collected samples were not used in this study.
Ethics oversight	Experiments involving mice were performed according to protocols approved by the Stanford University Institutional Care and Use Committee (IACUC)

Note that full information on the approval of the study protocol must also be provided in the manuscript.

Plants

Seed stocks	Not applicable
Novel plant genotypes	Not applicable
Authentication	Not applicable
